# Deciphering the role of LOC124905135-related non-coding RNA cluster in human cancers: A comprehensive review

**DOI:** 10.1016/j.heliyon.2024.e39931

**Published:** 2024-10-31

**Authors:** Maryam Eftekhari Kenzerki, Amirhossein Mohajeri Khorasani, Iman Zare, Farzane Amirmahani, Younes Ghasemi, Michael R. Hamblin, Pegah Mousavi

**Affiliations:** a Department of Medical Genetics, School of Medicine, Tehran University of Medical Sciences, Tehran, Iran; b Department of Medical Genetics, Faculty of Medicine, Hormozgan University of Medical Sciences, Bandar Abbas, Iran; c Student Research Committee, Hormozgan University of Medical Sciences, Bandar Abbas, Iran; d Research and Development Department, Sina Medical Biochemistry Technologies Co., Ltd., Shiraz, 7178795844, Iran; e Department of Cell and Molecular Biology and Microbiology, Faculty of Science and Technology, University of Isfahan, Isfahan, Iran; f Pharmaceutical Sciences Research Center, Shiraz University of Medical Sciences, Shiraz, Iran; g Department of Pharmaceutical Biotechnology, School of Pharmacy and Pharmaceutical Sciences Research Center, Shiraz University of Medical Sciences, Shiraz, Iran; h Laser Research Centre, Faculty of Health Science, University of Johannesburg, Doornfontein, 2028, South Africa; i Radiation Biology Research Center, Iran University of Medical Sciences, Tehran, Iran; j Molecular Medicine Research Center, Hormozgan Health Institute, Hormozgan University of Medical Sciences, Bandar Abbas, Iran

**Keywords:** LOC124905135, Long non-coding RNAs, MicroRNAs, Prognosis, Oncogenes, Tumor suppressor gene

## Abstract

Non-coding RNAs (ncRNAs), especially microRNAs (miRNAs) and long ncRNAs (lncRNAs), are essential regulators of processes, such as the cell cycle and apoptosis. In addition to interacting with intracellular complexes and participating in diverse molecular pathways, ncRNAs can be used as clinical diagnostic biomarkers and therapeutic targets for fighting cancer. Studying ncRNA gene clusters is crucial for understanding their role in cancer and developing new treatments. LOC124905135 is a protein-coding gene encoding a collagen alpha-1(III) chain-like protein, and also acts as a gene for several ncRNAs, including miR-3619, PRR34 antisense RNA 1 (PRR34-AS1), PRR34, long intergenic ncRNA 2939 (LINC02939), LOC112268288, and MIRLET7BHG. It also serves as a host gene for three miRNAs (hsa-let7-A3, hsa-miR-4763, and hsa-let-7b). Notably, the ncRNAs derived from this particular genomic region significantly affect various cell functions, including the cell cycle and apoptosis. This cluster of ncRNAs is dysregulated in several types of cancer, exhibiting mutations, alterations in copy number, and being subject to DNA methylation and histone modification. In summary, the ncRNAs derived from the LOC124905135 cluster could be used as targets for diagnosis, therapy monitoring, and drug discovery in human cancers.

## 1 Introduction

Cancer is the most common cause of death all around the world after cardiovascular disease and affects about 8 million people annually. Cancer prevalence has been predicted to increase by 50 % in future decades [1]. Identifying regulatory mechanisms can aid in developing accurate diagnostic tools and treatment options for malignancies [2]. Many factors are involved in transforming a normal, mature, differentiated cell into a fast-growing, aggressive, and apoptosis-resistant cell known as a malignant cell. Cancer cells exhibit eight key features called hallmarks, including increased proliferative signaling, evasion of growth repressors, inhibition of apoptosis, never-ending replication, inducing and accessing vasculature, increased invasion and metastasis, reprogramming of cellular metabolism, and avoidance of immune attack [3].

Regulatory RNAs play vital roles in many cell biology-related and biomedical processes. NcRNAs can control diverse biological functions and networks during both normal development and in pathological diseases. They account for over 60 % of the total gene transcription in human cells. Various ncRNA classes, such as lncRNAs, miRNAs, circular RNAs (circRNAs), small interfering RNAs (siRNAs), and others that are still being identified or explored have been described [4]. The interplay between RNA-binding proteins (RBP) and ncRNAs regulates the translation of mRNAs into functional proteins [5]. LncRNAs are functional biomolecules that cooperate with other cell components such as DNA, proteins, or RNAs, to regulate gene expression [6]. LncRNAs can directly affect gene expression by altering histone methylation and acetylation through epigenetic changes or can act as a scaffold for protein complexes that can modify histones and influence gene expression [7]. Moreover, lncRNAs can act as micro-RNA sponges to control the expression of specific cellular proteins [8]. The lncRNA complex comprises numerous proteins and RNA molecules that cooperate to promote mRNA destruction, translational suppression, and nucleosome structural reconfiguration based on the mRNA complementary sequences [9].

The term “gene cluster” refers to a set of genes situated nearby on a chromosome, with the genes in question working in tandem or carrying out similar functions. The study of gene clusters has played a critical role in elucidating genome's function and in evolution. However, existing knowledge about gene clusters primarily centers on protein-coding genes, with less attention being paid to ncRNA clusters that also exhibit clustered arrangements. Gene clusters based on ncRNAs could be a valuable resource for deepening our understanding of these RNAs in the context of evolution and human disease, particularly cancer. Nonetheless, only recently have researchers begun to explore these ncRNA clusters, thereby opening up new avenues for investigating the underlying mechanisms of gene regulation [10,11]. The ncRNAs originating from LOC124905135 and clustered in the 22q13.31 region have been the subject of several investigations lately, revealing their significant role in the progression of different types of cancer, acting either as oncogenes or tumor suppressors [[12], [13], [14], [15]]. This review aims to offer a comprehensive insight into the function and mechanisms of LOC124905135-derived ncRNAs in cancer development and progression.

## 2 Non-coding RNAs

miRNAs are a class of small ncRNA molecules that are ubiquitous in animals and plants. They are typically 19–25 nucleotides in length and bind to the 3′ untranslated region (UTR) of their target mRNAs, leading to translational repression or mRNA degradation. Due to their regulatory roles, miRNAs are involved in many biological processes, including normal development and differentiation, as well as disease pathogenesis. Their small size and high abundance make them attractive biomarkers for the diagnosis and prognosis of diseases, as well as potential therapeutic targets for drug development [16,17]. Human miRNAs are usually located within gene introns or ncRNA transcript regions, and they can regulate the expression of around 30 % of human protein-coding genes [18]. Due to the short sequence of the duplex miRNA-3′UTR binding region, a single miRNA can interact with tens or even hundreds of different target mRNAs, or else a single specific mRNA can be regulated by multiple miRNAs. Ultimately, targeted mRNAs are typically destroyed or prevented from being translated. miRNA-encoding genes may be co-expressed and controlled by a single regulatory element within a miRNA cluster. The components of a miRNA cluster can target the same gene or separate genes [19]. Therefore, several miRNAs can form a complex network (cluster miRNAs), and their downstream genes can participate in various cell processes such as early development, proliferation, differentiation, and apoptosis [20,21].

The regulation of miRNA cluster gene expression involves both genetic and epigenetic mechanisms, similar to the regulation of protein-coding genes. miRNA clusters can control the proliferation, differentiation, metabolic pathways, immunological response, cellular damage, organelle biogenesis, signal transduction, and DNA repair of normal cells as well as cancer cells. The disruption of miRNA clusters, resulting in disturbed cellular functions, is important in the pathophysiology of several diseases, particularly cancer [22]. Several studies have indicated that miRNAs perform important functions in tumor initiation, growth, and therapeutic response. Fragile sites or genomic regions are enriched in about 50 % of miRNAs isolated from tumors, underlying their probable critical role in tumorigenesis [23]. Advanced tumors are associated with abnormally expressed miRNAs that can interact directly or indirectly with oncogenes or tumor suppressor genes to regulate their expression. Therefore, miRNAs can act as promising tumor biomarkers and may be targets for cutting-edge anticancer therapies due to their function as either tumor suppressors or as oncogenes [24].

LncRNAs, like miRNAs, are recognized regulators of gene expression in various biological settings [25]. They are defined as ≥200 nucleotide ncRNAs to distinguish them from small ncRNAs [26]. LncRNAs are classified into intergenic (between protein-coding genes; long intergenic ncRNA [lincRNA]), intronic, and natural antisense transcripts (NATs), or else can be transcribed from different enhancers and promoters [27]. Certain aspects of lncRNA biology are similar to mRNAs, as both sequences are transcribed by RNA polymerase II, followed by capping and polyadenylation [28]. LncRNAs are tightly controlled in a reverse direction, localized in different subcellular compartments, and eventually carry out diverse functions in human cells. The conserved sequence of a lncRNA does not always translate into conserved functions, and its processing and binding sites can significantly influence its subcellular distribution. Consequently, the localization of any specific lncRNA may significantly affect its function [29]. For example, circRNAs could be found in various subcellular fractions, with the exception of the mitochondria. The final location and function of circRNAs are determined by factors such as their length, GC content, parental gene function, and RBP-mediated selective transportation [30,31]. While lncRNA expression levels are typically lower than mRNAs, they are more likely to be expressed in solid tumors, suggesting their vital role in cell type-specific processes such as the cell cycle, cellular differentiation, metabolism, and carcinogenesis [29,[32], [33], [34]].

One mechanism by which lncRNAs regulate gene expression is through the sequestration of complementary miRNAs, thereby inhibiting the ability of miRNAs to bind to the 3′UTR of target mRNAs and prevent their degradation, as outlined by Zang et al. [35]. LncRNAs are also able to bind to gene enhancers, thus affecting gene expression leading to adverse effects in various human diseases [36,37]. Additionally, They can interact with transcription factors in multiple cancers [10]. LncRNAs were previously thought to be functional chromatin regulators, leading to the assumption that they generally belonged in the nucleus [38,39]. The cytoplasmic role of lncRNAs is to mediate signal transduction pathways, control translation, and posttranscriptional regulation of gene expression. LncRNAs interact with both miRNAs and proteins, modulating their function and expression levels through various mechanisms, including promoting post-translational modification of proteins, facilitating mRNA translation, and enhancing mRNA stability [[40], [41], [42]]. Furthermore, many mitochondrial lncRNAs have been implicated in the regulation of mitochondrial genes, while some nuclear-transcribed lncRNAs play vital roles in maintaining mitochondrial homeostasis [43,44]. The presence of single nucleotide polymorphisms (SNPs) in or near the lncRNA sequence, as well as single nucleotide variants (SNVs) within lncRNAs, can significantly impact the expression of lncRNAs. Consequently, genetic variations that could alter lncRNA expression might influence their downstream gene regulation, potentially leading to cancer progression [45].

## 3 LOC124905135-derived ncRNAs

Based on NCBI (National Center for Biotechnology Information) information, a protein-coding gene called *LOC124905135* encodes a known protein, the collagen alpha-1(III) chain, derived from a genomic sequence (NC_000022.11, located at chr22:46044644.46113928). This sequence has been annotated using the highly reliable gene prediction method called “Gnomon”, and is supported with mRNA and EST evidence to ensure its accuracy and validity. *LOC124905135* contains four coding transcript variants, comprising X1 variant (XM_047441694), X2 variant (XM_047441695), X3 variant (XM_047441696), and X4 variant (XM_047441697) producing linear mRNAs with lengths of 16167 bp, 16937 bp, 16367 bp, and 16369 bp, respectively (https://www.ncbi.nlm.nih.gov/nuccore/?term=LOC124905135).

In addition, this particular gene serves as a host gene for multiple ncRNAs, including miR-3619, PRR34 antisense RNA 1 (PRR34-AS1), PRR34, long intergenic ncRNA 2939 (LINC02939), LOC112268288, and MIRLET7BHG. On the other hand, MIRLET7BHG acts as a host gene for three miRNAs (has-let7-A3, has-miR-4763, and hsa-let-7b).

Additional information about LOC124905135-derived ncRNAs based on the NCBI database is as follows:

### 3.1 PRR34

PRR34 is a 5695 nt lncRNA that is found at chr22:46,048,531–46,054,225. It has a chain length of 3845 nucleotides and is encoded by the 22q13.31 locus from the source gene LOC124905135 (https://www.ncbi.nlm.nih.gov/nuccore/1790132980).

### 3.2 PRR34-AS1

PRR34-AS1 is located at chr22:46,053,846 - 46,058,522 with a length of 4677 nt. It has a chain length of 1635 nucleotides with NCBI Reference Sequence of NR_027034.1 and is encoded by the 22q13.31 region from the source gene of LOC124905135 (https://www.ncbi.nlm.nih.gov/nuccore/NR_027034.1).

### 3.3 LINC02939

LINC02939 is a 6371 nt lncRNA located at chr22:46,064,651–46,071,021 position. It has a total RNA chain length of 3604 nucleotides with the accession number NR 148973.1 and is transcribed by the 22q13.31 locus from the source gene of LOC124905135 (https://www.ncbi.nlm.nih.gov/nuccore/1237938463).

### 3.4 MIRLET7BHG

MIRLET7BHG is a lncRNA (NC_000022.11, NC_060946.1) encoded by the 22q13.31 region. MIRLET7BHG (chr22:46,053,869–46,113,928), ENSG00000273289, and PRR34-AS1 were shown to be adjacent in the positive strand, and ENSG00000231010, LOC642648, and GC22M046089 in the negative strand by the Genome locator (https://www.ncbi.nlm.nih.gov/gene/400931). It has two non-coding transcripts named NR_027033.2 with a length of 4717 nucleotides and NR_110479.1 with 4566 nucleotides (https://www.ncbi.nlm.nih.gov/nuccore?LinkName=gene_nuccore_refseqrna&from_uid=400931).

### 3.5 LOC112268288

LOC112268288 is a lncRNA with two variants known as X1 (XR_002958732.2), located at chr22:46,086,167–46,097,919 with 11,634 nt, and X2 (XR_002958733.2). This lncRNA is derived from LOC124905135 (https://www.ncbi.nlm.nih.gov/gene/124905135).

### 3.6 Hsa-miR-3619

hsa-miR-3619 is a linear microRNA with the NR_037413 accession number in the NCBI database (https://www.ncbi.nlm.nih.gov/gene/?term=hsa-mir-3619). It is located at chr22:46,091,044–46,091,126 with a length of 83 nucleotides, consisting of two sequences: hsa-miR-3619-5p (miRBase: MIMAT0017999) and has-mir-3619-3p (miRBase: MIMAT0019219).

### 3.7 Hsa-let-7a-3

hsa-let-7a-3*,* also known as LET7A3, let-7a-3, and MIRNLET7A3, is another ncRNA with a length of 74 nucleotides derived from the LOC124905135 gene. It is encoded by chr22:46,112,749–46,112,822 and has two variants called the miR-let7-a3-3p and the miR-let7-a3-5p (https://www.ncbi.nlm.nih.gov/gene/406883).

### 3.8 Hsa-miR-4763

hsa-miR-4763 is a 92-nucleotide-long microRNA located in chr22:46,113,566–46,113,657 has two variants, namely hsa-miR-4763-3p and hsa-miR-4763-5p (https://www.ncbi.nlm.nih.gov/gene/?term=MiR-4763).

### 3.9 Hsa-let-7b

hsa-let-7b is the final ncRNA that is encoded by the MIRLET7BHG gene. This well-known microRNA, located at chr22:46,113,686–46,113,768 with a length of 83 nucleotides, is dysregulated in many kinds of human cancer and has two variants, including hsa-let-7b-3p and hsa-let-7b-5p (https://www.ncbi.nlm.nih.gov/gene/406884).

**Fig. 1:**
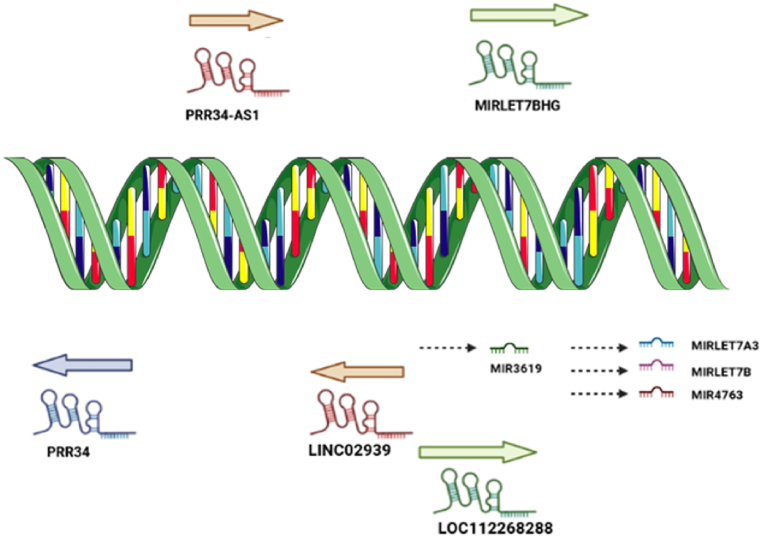
The RNA cluster LOC124905135 is a host gene for several non-coding RNAs. LOC124905135 is the source of five lncRNAs: three of them PRR34-AS1, MIRLET7BHG, and LOC112268288 are encoded by the positive-strand, while two of them PRR34 and LINC02939 are encoded by the negative strand of DNA. Furthermore, MIRLET7BHG itself leads to four microRNAs that play prominent roles in different cellular pathways and regulate various proteins. The first is miR-3619 which is released from the 3′UTR; while the last three miRNAs are miR-4763, miR-let7a3, and miR-let 7b which are released from the 5′UTR of MIRLET7BHG. Figure created with BioRender.com and smart.servier.com.

A summary of the biogenesis of ncRNAs derived from LOC124905135 is shown in Fig. 1.

**Fig. 2:**
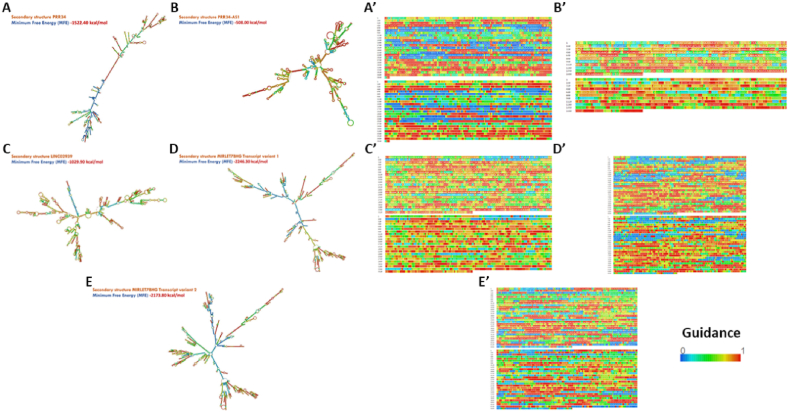
Prediction of the optimum secondary structure of the five LOC124905135-derived ncRNAs. Forena format with a dot–bracket representation utilizing the Rfold online tool. In this structure, the color of the four ribonucleotides is: A, yellow; U, blue; C, green; G, red. Optimal secondary structure of **A)** PRR34 (Forena format) with −1522.40 kcal/mol, **B)** PRR34-AS1 (Forena format) with −508.00 kcal/mol, **C)** LINC02939 (Forena format) with −1029.90 kcal/mol, **D)** MIRLET7BHG variant 1 (Forena format) with −2246.30 kcal/mol, and **E)** MIRLET7BHG variant 2 (Forena format) with −2173.80 kcal/mol. Dot–bracket representation of **A׳)** PRR34, **B׳)** PRR34-AS1, **C׳)** LINC02939, **D׳)** MIRLET7BHG variant 1, and **E׳)** MIRLET7BHG variant 2 (For interpretation of the references to color in this figure legend, the reader is referred to the Web version of this article).

The mfold web server was utilized (http://rna.tbi.univie.ac.at/cgi-bin/RNAWebSuite/RNAfold.cgi) to construct an optimal secondary structure prediction of the PRR34-AS1, PRR34, LINC02939, LOC112268288, and MIRLET7BHG, with minimum free energy (MFE). The result is shown in Fig. 2 (A-E^׳^) [46,47].

## 4 Protein-RNA interactions and cellular networks related to LOC124905135-derived non-coding RNAs

Many molecular mechanisms involving cancer cell communications and the tumor microenvironment remain unknown. Cancer tissue or a tumor mass is composed of malignant cells with different pathological features, and is considered a complication of cancer tissue. Cancer heterogeneity poses a significant obstacle to understanding the molecular pathways underlying oncogene functions. Tissue heterogeneity is a complex biological process in some malignancies, referring to the altered expression and function of hub genes (mRNAs and ncRNAs) in a regulatory network that affects many downstream signaling pathways [48]. Finding ways to simplify the complex interactions and dysregulation of protein expression levels could be useful in treating tumors and reducing recurrence.

### 4.1 PRR34-AS1

PRR34-AS1 is significantly overexpressed in various cancers, such as hepatocellular carcinoma (HCC) and acute myeloid leukemia (AML). PRR34-AS1 directly targets miR-498 by sponging, which results in an increased expression of the transcription factor called fork-head box O3 (FOXO3) in HCC cells. Therefore, the PRR34-AS1/miR-498/FOXO3 axis can play a crucial role in HCC [49]. PRR34-AS1 lncRNA increased E2F2 and SOX12 expression by sponging miR-296–5p in HCC cells, thus stimulating the Wnt/β-catenin pathway and promoting cell proliferation and tumor progression [50]. PRR34-AS1, TOMM20, and ITGA6 were remarkably up-regulated in progressing HCC tumors. As cancer progresses, the expression level of miR-498 is markedly reduced, indicating its ability to inhibit cancer cell proliferation, invasion, and migration [51]. Also, PRR34-AS1 promotes the progression of HCC by acting as a sponge for miR-296-5p. This interaction modulates the expression of E2F2 and SOX12 positively. E2F2 activates the transcription of PRR34-AS1, which targets the miR-296-5p/E2F2/SOX12/Wnt/β-catenin axis to promote HCC progression. An *in vitro* and *in vivo* study found that PRR34-AS1 showed high expression in HCC cells, which indicated its involvement in HCC cell properties, including proliferation, migration, and invasion (PMI) and growth, and the epithelial-mesenchymal transition (EMT) process [52]. Consequently, PRR34-AS1 can be considered a potential oncogene that is up-regulated in many types of cancer and plays a role as an oncogenic transcription factor to promote tumor cell proliferation and progression.

### 4.2 MIRLET7BHG

Various human malignancies exhibit aberrant expression of the lncRNA MIRLET7BHG, which is overexpressed in the malignant tissue samples and cell lines of 12 different forms of human cancer, including AML [53], basal-like breast and ovarian cancer (OVC), colon cancer [54], colorectal cancer (CRC) [55], esophageal adenocarcinoma and lung cancer [[56], [57], [58], [59]]. It is also implicated in HCC [60,61], embryonal brain tumors (embryonal tumor with multilayered rosettes, ETMR) [62,63], glioma [64], pancreatic cancer [65], bladder cancer [66], and melanoma [67]. Evaluation of Cancer Genome Atlas (TCGA) gene expression data from the Genomics Data Collaborative (GDC) in the TCGA-AML cohort of 151 patients revealed higher levels of MIRLET7BHG expression in AML tissues compared to normal controls [53]. By analyzing 446 paired colon samples from the TCGA database, a correlation was found between high expression of MIRLET7BHG and poor overall survival (OS) in colon cancer patients [54]. In contrast, MIRLET7BHG was significantly decreased in pancreatic ductal adenocarcinomas (PDAC), as observed in TCGA-Pancreatic Adenocarcinoma (PAAD) subtypes (84 classical and 65 basal-like) and the Genotype-Tissue Expression (GTEx) project (248 normal pancreas samples) [65].

**Fig. 3:**
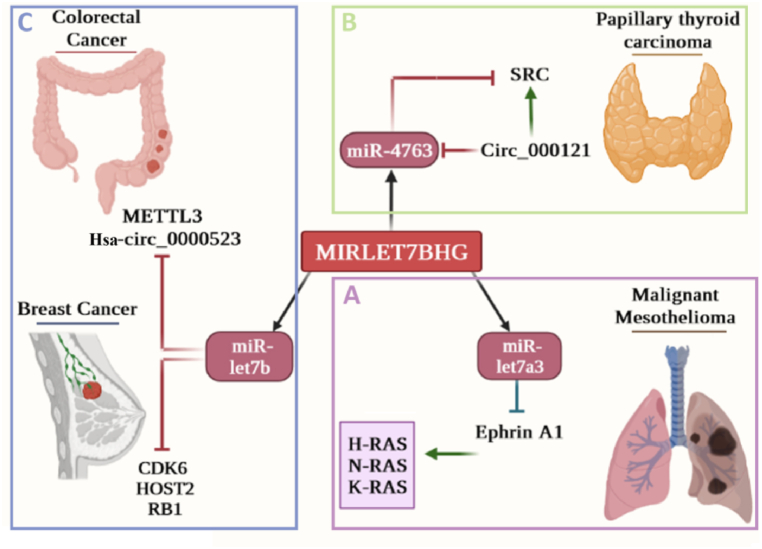
The probable role of MIRLET7BHG in different cancers. A) Ephrin A1 is a tyrosine kinase receptor that is crucial for cellular migration, repulsion, and adhesion during neuronal, vascular, and epithelial development. It increases H/N/K-RAS levels in cells, especially in mesothelioma cancer. MiR-let7a3 reduces the Ephrin A1 levels in cells. **B)** In papillary thyroid carcinoma, SRC (non-receptor tyrosine kinase) is reduced by miR-4763, while it can be enhanced by Circ-000121 through sponging miR-4763. **C)** miR-let7b may play a role in breast and colorectal cancer by controlling important proteins, such as CDK6, RB1, HOST2, and METTLE3. Created with BioRender.com.

In the development of gastric cancer (GC), the abnormal overexpression of the transcription factor KLF5 results in the dysregulation of several lncRNAs, such as MIRLET7BHG, leading to the disruption of the normal function of several miRNAs (hsa-miR-873, hsa-miR-522, hsa-miR-505, hsa-miR-484, hsa-miR-374a, hsa-miR-340, hsa-miR-331, and hsa-miR-151a) and thus promotes cancer growth through a ceRNA network. In non-atrophic gastritis, the levels of MIRLET7BHG were significantly decreased during pre-cancerous stages but remained present in the later stages of disease development [68]. Cross-linking and immunoprecipitation (CLIP), as well as complementary and proximity-based methods, have been employed to identify specific RNA-binding sites on endogenous RNAs [69]. The AGO2-CLIP immunoprecipitation technique confirmed that lncRNA MIRLET7BHG is bound to the essential RISC protein, with cross-linking peaks detected at sites representing mature miRNAs. LIN28B inhibited let-7b by regulating AGO2 in the RISC complex. TEAD2, a transcription factor, plays a significant role in the Hippo signaling pathway by networking with YAP1, which binds and regulates MIRLET7BHG, leading to decreased basal-like tumor growth in pancreatic cancer [65,[70], [71], [72]]. Therefore, MIRLET7BHG can be considered a promoter of cancer cell proliferation because of its role as a host gene for some important ncRNAs. The probable role of MIRLET7BHG in different cancers is summarized in Fig. 3.

### 4.3 miR-3619

Hsa-miR-3619-5p is down-regulated in various malignancies, such as HCC, retinoblastoma (RB), GC, and squamous-cell carcinoma [[73], [74], [75], [76]]. Breast cancer cell lines (MCF-7 and T47D) exhibited a significant decrease in p21 and miR3619-5p levels, along with an increase in cyclin D1 and cyclin-dependent kinase 6 (CDK6) expression. After transfection of miR-3619-5p into MCF-7 and T47D cells, it was found that miR3619-5p overexpression could effectively inhibit breast cancer cell proliferation and promote apoptosis by activating p21 expression in breast cancer cells [77]. In EJ and T24 bladder cancer cells, miR-3619-5p promoted p21 transcription by binding to its promoter. The induced transcription of miR-3619-5p consistently resulted in β-catenin and CDK2 down-regulation via binding to their 3′UTRs in a p21-dependent manner, thus preventing the EMT [78]. Circ_0026134 and CHAF1B are highly expressed in NSCLC tissues and cell lines, promoting NSCLC cell progression. Circ_0026134 can sponge miR-3619-5p, a regulator of CHAF1B, suggesting that the Circ_0026134/miR-3619-5p/CHAF1B axis could function as a predictor of NSCLC behavior *in vitro* [13]. PVT1 acts as a competing endogenous RNA (ceRNA) by decoying miR-3619-5p and destroying TRIM29 in CRC [79]. Circ_0011292 targets miR-3619-5p, an important mechanism in PTX-resistant NSCLC cells, resulting in the down-regulation of CDCA4, thus inhibiting apoptosis while simultaneously enhancing cell proliferation and invasion [80].

**Fig. 4:**
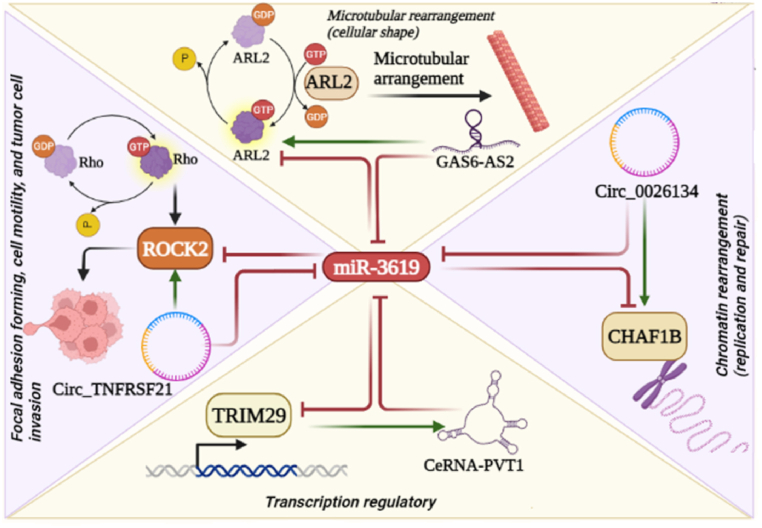
Roles of miR-3619 as an expression regulator in cells. ARL2 (Arf family of regulatory GTPases) plays a significant role in cellular morphology and structure via microtubular arrangement, and can be targeted by miR-3619. Moreover, ARL2 is enhanced by GAS6-AS2 by sponging miR-3619. CHAF1B is the p60 subunit of chromatin assembly complex-1 (CAF-1), and is also the primary chaperone of a group of histone-like proteins called protamines, which are small highly basic arginine-rich linker proteins that share a high degree of homology with histone linker protein H1. CHAF1B is important in replication and repair-dependent nucleosome assembly, and can be targeted and inhibited by miR-3619, while Circ_0026134 sponges miR-3619 and enhances CHAF1B expression. TRIM29 is a protein with multiple zinc finger motifs plus a leucine zipper motif that enables it to act as a transcription factor and is also regulated by miR-3619. CeRNA-PVT1 up-regulates TRIM29 by sponging miR-3619. ROCK2 (Rho-associated coiled-coil kinase-2) is a serine/threonine kinase activated by Rho GTPases. ROCK2 can modulate cytoskeletal rearrangement by forming focal adhesions, thus affecting cell motility and invasion in tumor cells. As shown in panel “d” ROCK2 can be reduced by miR-3619 and increased by Circ-TNFRSF21 which sponges miR-3619. Created with BioRender.com.

Circ_TNFRSF21 was up-regulated in M2 macrophages and also mediated M2 macrophage-induced endothelial cell tube formation by sponging miR-3619-5p to up-regulate ROCK2 [81]. In stomach adenocarcinoma (STAD), miR-3619-5p expression was conspicuously down-regulated in cells and tissues. Tumor properties, including PMI and growth, were suppressed, while tumor progression was increased by miR-3619-5 through the down-regulation of its downstream gene, TBC1D10B. Moreover, STAT4, by binding to the miR-3619-5p promoter, inhibited its transcription [82]. When Huh7-H and MHCC97-H cells were heat treated *in vitro* as a model of insufficient radiofrequency ablation (RFA) in HCC tumors, GAS6-AS2 acted as a ceRNA by sponging miR-3619-5p and regulating ARL2 (ADP ribosylation factor-like GTPase 2) [83]. PVT1 acted as a sponge for miR-3619-5p, increasing MKL1 expression and migration in HCC cells [84]. Also, overexpression of HCP5 in GC cells cultured with mesenchymal stem cells increased 5-fluorouracil and oxaliplatin resistance by regulation of PPARGC1A-mediated FAO by decoying miR-3619-5p [85]. miR-3619 is a potential regulator of malignant pathways by inhibiting the expression of critical genes involved in invasion and proliferation. Some aspects of how miR-3619 functions as a regulator of gene expression in cells are illustrated in Fig. 4.

### 4.4 Let-7a-3

Ephrin A1 activation increases the expression of let7-a3 miRNA and reduces the expression of H-RAS, K-RAS, and N-RAS, thus inhibiting cancer growth [86]. The miRNA let-7a-3 is up-regulated in some cancer types and appears to promote the proliferation and progression of tumor cells.

### 4.5 miR-4763

The up-regulation of hsa-miR-4763-3p was found to be significant in peripheral T-cell lymphoma, not otherwise specified (PTCL-NOS) [87]. The expression of miR-4763 was increased in the peripheral blood cells of patients with non-metastatic papillary thyroid microcarcinoma (PTMC). Moreover, hsa_circRNA_000121 may up-regulate SRC (sarcoma gene) expression by modulating hsa-miR-4763 [88]. ncRNA BOC contains potential sites for hsa-miR-4763-5P binding, suggesting its role in glioblastoma progression through miRNA sponging of downstream genes [89]. In the MAPK signaling pathway, hsa-miR-4763-3p targets NFATC4 and JUND [90]. miR-4763-5p seems to have a critical function in controlling tumor cell pathways and promoting proliferation and progression.

### 4.6 Let-7b

Let-7b was shown to be down-regulated in various cancers, such as prostate cancer [14], breast cancer [91], and NSCLC [92]. Several molecular biology methods have shown that has_circ_0000523 indirectly regulates the expression of METTL3 by inhibiting the transcription of miR-let7b [93]. Let-7b, a downstream target of lncRNA HOST2, binds directly to the 3′UTR of CDK6, effectively repressing its expression. This discovery highlights the crucial role of let-7b in regulating CDK6 and underscores the importance of understanding the intricate mechanisms that govern gene expression [94]. Furthermore, there was a negative correlation between NEAT1 lncRNA and let-7b in HCC tissues. NEAT1 promoted HCC cell proliferation and reduced apoptosis in HepG2/Huh7 cell lines by regulating the Let-7b-IGF-1R Axis [95]. Considering its multifaceted effects on proliferation and progression, miR-let-7b is a potent tumor suppressor that can regulate various critical cellular pathways. Its ability to modulate diverse pathways ultimately leads to the inhibition of tumor growth and progression, making it a crucial therapeutic target in cancer research.

## 5 Methylation and histone modification status of LOC124905135 derived non-coding RNAs

Epigenetic modifications mainly involve DNA methylation and histone modification, which alter DNA transcription and chromatin structure to regulate gene expression. Epigenetic processing is critical for normal development and the differentiation of various cell lineages in the adult organism. Various histone modifications regulate many cellular processes, such as gene transcription, replication, and repair, in a synchronized and choreographed manner. Histone-modification complexes change the pattern and levels of histone methyl and acetyl groups, controlling chromatin-based expression processes. As a result, DNA and histone binding proteins, as well as ncRNA molecules, play an essential role in chromatin remodeling, which can lead to oncogenic changes and the progression of human tumors [96,97]. In some cases, genes encoding transcription factors and cascade mediator proteins that might behave as oncogenes can be hypomethylated, resulting in overexpression of cancer progression driver genes.

### 5.1 PRR34-AS1

Using methylation-specific PCR (MSP) and bisulfite sequencing PCR, Nan et al. discovered a hypomethylation pattern in the promoter of PRR34-AS1 in AML patients compared to normal controls. The promoter hypomethylation pattern and the increased expression of PRR34-AS1 may indicate a poor prognosis with a shorter OS in AML and non-APL-AML patients [98].

### 5.2 MIRLET7BHG

MIRLET7BHG is up-regulated in MDS and AML CD34^+^ cells. It decreased EZH2 and KDM2B protein levels by affecting H3K27me3 and cyclin D1, reducing S-phase cells, and increasing G0/G1 cells [99]. Hypomethylation of the MIRLET7BHG promoter can lead to an increase in its expression and the processing of its specific miRNAs in various malignancies.

### 5.3 miR-3619

RB tissues and cell lines show increased levels of HCP5 and HDAC9, while miR-3619-5p levels are decreased. Experimental studies using cell culture and animal models have demonstrated that down-regulation of HCP5 or up-regulation of miR-3619-5p can effectively inhibit cancer progression, particularly proliferation and invasion, and vice versa [100]. LINC01535 inhibits human bone marrow stem cell (hBMSC) proliferation, osteogenic differentiation, and apoptosis by controlling BMP2 expression levels through sponging miR-3619-5p [101]. The miR-3619 is a potential tumor suppressor that can control the expression level of several mRNAs, such as histone deacetylases and transcription factors, thereby controlling cell cycle and signal transduction pathways.

### 5.4 Let-7a-3

A miRNA's epigenetic dysregulation can disrupt several potential downstream pathways of its targets; for instance, hypermethylation of the let7a-3 promoter leads to cell proliferation and progression in OVC [102]. The let-7a-3 gene, located near a CpG island on chromosome 22q13.31, can be methylated by DNMT1 and DNMT3B. In normal human tissues, it is highly methylated. However, in some lung adenocarcinomas, it is hypomethylated, leading to increased expression and promoting tumor growth in human lung cancer cell lines [103]. Hypermethylation of the MIRLRT7A3 gene down-regulates miR-let-7a microRNA in HCC cells, promoting the oncogenic IGF-signaling pathway through increased expression of IGF-II and IGF2BP-2/-3. DNA-methyltransferase inhibitors can restore miR-let-7a expression, reducing IGF expression by binding to their specific transcripts [104]. let-7a-3 is an epigenetically regulated microRNA gene with dual functions, acting as either an oncogene or a tumor suppressor. Its role in human cancer may involve contributions to the cancer epigenome through aberrant miRNA methylation.

### 5.5 Let-7b

Alterations in DNA methylation patterns of super-enhancers in human malignancies are associated with either transcriptional silencing or hyperactivation of their target genes [105]. According to an in silico study, inhibition of tumor suppressor let-7b and let-7a-3 in breast and lung cancers can be associated with the super-enhancer controller lncRNA MIRLET7BHG [106]. Furthermore, KDM2B inhibited the miR-let-7b-5p transcript via H3K36me2 epigenetic modification, boosting the transcription level of enhancer of zester homolog-2 (EZH2), a specific target gene of miR-let-7b-5p in NSCLC. EZH2 increased the transcriptional level of PKMYT1 to activate the Wnt/-catenin pathway. Sh-EZH2 and sh-PKMYT1 inhibited KDM2B's beneficial effects on cell proliferation, invasion, and migration. The KDM2B affects the EZH2/PKMYT1/Wnt/β-catenin axis by preventing the let-7b-5p expression [107,108]. Consequently, dysregulation of several histone demethylases in various cancer types leads to the overexpression of distinct oncogenes, thereby causing a reduction in miR-let-7b expression.

## 6 LOC124905135-derived non-coding RNAs, inflammation, and drug resistance

Inflammation, particularly the activation of NF-κB and its associated genes, has been linked to cancer. NF-κB activation leads to increased levels of IL6, which mediates the activation of the transcription factor STAT3. Let-7 directly regulates IL6 expression, completing a positive feedback loop. This regulatory circuitry is observed in various tumor cell lines and is evident in human tumor tissues. Inflammation initiates a self-sustaining positive feedback loop, maintaining the epigenetically transformed state for multiple generations even without the inducing stimulus [109].

Drug resistance in cancer cells can occur due to various causes, such as genetic variations, inhibition of cell death, modification to drug metabolism, epigenetic changes, gene amplification, and DNA repair processes. The development of drug resistance is often due to alterations in drug targets. However, advances in targeted therapy due to DNA microarray and proteomics technology have presented new opportunities to combat drug resistance. Despite progress in designing new chemotherapy agents against advanced stages of cancer, combating invasion and metastasis remains challenging [110]. Hence, this section aims to clarify correlations between ncRNAs derived from LOC124905135 and their potential involvement in drug resistance and inflammation. Addressing these molecular networks can provide promising insight into the complex mechanisms that underlie drug resistance and inflammation in cancer.

### 6.1 PRR34-AS1

The FOXO subfamily of fork-head transcription factors is largely influenced by cancer progression and drug resistance. PRR34-AS1 increases FOXO3a expression by sponging miR-498, thus promoting drug resistance and cancer progression [111].

### 6.2 MIRLET7BHG

The TCGA database, a developed model for predicting esophageal adenocarcinoma, can be used to determine the expression levels of 12 immune-related lncRNAs, one of which is MIRLET7BHG, which is considered as a promising prognostic biomarker [112].

### 6.3 miR-3619

IL-6, TNF-α, ACAN, and COL2A1 cause inflammation and degradation of the ECM. SIRT1 reduces ACAN and COL2A1 while increasing IL-6, TNF-α, and ADAMTS5. Circ_0022383 acts as a sponge for miR-3619-5p, which targets ADAMTS5, thus reducing inflammation, programmed cell death (PCD), and ECM degeneration [113]. The pathway involving Notch1 and lncNDEPD1 plays a key role in regulating PD-1 expression in human CD8^+^ T cells. LncNDEPD1 binds to miR-3619-5p, which in turn binds to PDCD1 mRNA, thereby preventing its destruction and increasing PD-1 expression. The direct binding of Notch1 to the lncNDEPD1 promoter further enhances this pathway. In addition, chimeric antigen receptor T cells (CAR T-cell) with low levels of lncNDEPD1 expression show more tumoricidal activity in the presence of PD-L1 [114]. MiR-3619-5p plays a crucial role in inhibiting cell proliferation and increasing drug sensitivity in cisplatin-resistant cutaneous squamous cell carcinoma (CSCC) cells by targeting and suppressing the expression of karyopherin subunit alpha 4 (importin alpha 3, KPNA4), which is responsible for the nuclear translocation of many transcription factors [75].

LncRNA HCP5 is implicated in the increased expression of peroxisome proliferator-activated receptor gamma (PPARγ) coactivator 1 alpha (PPARγC1A), and activation of the PPAR coactivator‐1α (PGC1α)/CEBPB pathway by blocking miR-3619-5p. LncRNA HCP5 stimulated fatty acid oxidation (FAO) in GC cells by transcriptionally activating carnitine palmitoyl-transferase 1 (CPT1). MSC-induced lncRNA HCP5 repressed FAO via the miR-3619-5p/AMPK/PGC1/CEBPB axis, promoting cell survival and chemoresistance in GC [73]. IL-6 activated STAT3, thus triggering stem cell-like properties in cisplatin-treated GC cells and up-regulating SNHG3 expression, positively correlated with cisplatin resistance and stemness. SNHG3 promoted oncogenic properties in GC cells by up-regulating ARL2 expression by miR-3619-5p sponging [115]. Exosomal HEIH boosted cisplatin resistance and apoptosis in tongue squamous cell carcinoma by decoying miR3169-5p, leading to up-regulation of HGDF (heparin-binding growth factor) [116]. Circ_TNFRSF21 down-regulates miR-3619-5p to promote M2 macrophage-mediated angiogenesis. In turn, miR-3619-5p suppresses angiogenesis by inhibiting ROCK signaling. Therefore, the circ_TNFRSF21/miR-3619-5p/ROCK2 axis can regulate M2 macrophage angiogenesis [81]. Circ 0011292 is highly expressed in PTX-resistant NSCLC cells, serving as a trap for miR-3619-5p, which targets CDCA4 in PTX-resistant NSCLC cells. Therefore, circ _0011292 can control CDCA4 expression by sponging miR-3619-5p [80]. MiR-3619-5p also plays a role in treatment resistance by sensitizing GC cells to (DDP) cisplatin by targeting TBL1XR1 [117].

### 6.4 Let-7a-3

According to microarray analysis, miRNAs such as let7a-3 could play a vital role in regulating neutrophil senescence, and the function of miRNAs may be a promising therapeutic approach for inflammatory diseases [118].

### 6.5 miR-4763

Multidrug-resistant GC cells were found to have a lower transcriptional level of miR-4763, suggesting the link between miR-4763 and GC drug resistance [119].

### 6.6 Let-7b

Treatment resistance has been associated with Let-7b down-regulation in various cancers, such as renal cell carcinoma [120], GC [121,122], HCC [123], OVC [124], and many others. Let-7b miRNA is down-regulated in MLL-rearranged ALL, and its regulatory region is hypermethylated in leukemic cells with an MLL fusion gene, but this can be restored by the epigenetic reversal agent 5-azacytidine [125]. Notch1 and the lncNDEPD1 axis regulate PD-1 expression in human CD8^+^ T cells. LncNDEPD1 is up-regulated in activated T cells and could prevent the increased expression of PD-1 by decoying miR-3619-5p. Notch1 binds to the lncNDEPD1 promoter, and CAR-T cells express lncNDEPD1-specific short hairpin RNAs, reducing its expression and increasing tumoricidal activity [114]. MiR-let-7b is reduced in chemoresistant OVC patients and is inversely correlated with COL3A1 levels. COL3A1 is linked to chemoresistance and is targeted by miR-let-7b. RRTS and taxol increase miR-let-7b expression, thus inhibiting proliferation in OVC cells [124].

The use of a miR let-7b-5p mimic in HepG2_HCC cells down-regulated IGF1R expression, inhibited the AKT/mTOR pathway, improved Ras/Raf signal transduction, and led to anti-tumor signaling in HCC xenograft mouse models by targeting GPC3 [126]. Treatment of PDAC is challenging due to a desmoplastic microenvironment that promotes tumor progression and blocks chemotherapy. The Hh signaling pathway supports desmoplasia and participates in PDAC initiation and progression. The Hh inhibitor GDC-0449 can suppress the Hh signaling pathway and restore miR-let7b expression for effective treatment. This combination treatment effectively suppressed tumor progression in mice compared to other treatments [127]. miR-let7b has a tumor suppressor effect in melanoma cells by regulating the cell cycle by targeting cyclin D. Curcumin, the active ingredient found in turmeric, has anti-cancer activity in melanoma and is well-tolerated by humans. Curcumin analogs like EF24 show improved anti-tumor efficacy and metabolic stability [128], highlighting the potential of miR-let7b in combating drug resistance and promoting anti-tumor activity.

## 7 LOC124905135-derived non-coding RNAs and autophagy

Autophagy is a multi-step cellular pathway activated under conditions of starvation, facilitating the removal of damaged or unnecessary proteins and organelles by transporting intracellular constituents to the lysosomes for degradation and subsequent recycling [129]. In practice, autophagy is essential for sustaining cell homeostasis and is strongly linked to the pathophysiology of various conditions [130]. To maintain homeostasis, the redeployment of intracellular autophagy-related degradation products can act as an alternative energy source during metabolic stress. Autophagy allows tumor cells to survive when apoptosis has failed. Defects in autophagy are associated with increased tumorigenesis, but the underlying mechanism is not well defined. It is thought that autophagy has a protective effect by lessening tumor necrosis and inflammation, thereby reducing DNA damage in tumor cells in response to metabolic stress [131]. Apoptosis-resistant human cancers are still subject to autophagy-mediated PCD. BECN1 protein (Beclin 1) is a crucial regulator of autophagic PCD, and the BECN1 gene acts as a tumor-suppressor haploinsufficient gene [132].

### 7.1 MIRLET7BHG

MIRLET7BHG has been identified as an autophagy-related lncRNA in AML, and its elevated expression levels have been linked to shorter patient survival rates [53]. In addition, MIRLET7BHG dysregulation in colon cancer has been linked with genomic instability and poor prognosis, triggering hepatic stellate cells via exosomal SMO, activating the Hedgehog pathway, and promoting the progression of HCC. For instance, Cao and colleagues suggested that MIRLET7BHG could interact with several miRNAs to target CTSD, BAX, and MAPK8IP1. Notably, CTSD and BAX have been implicated in the onset of nonalcoholic fatty liver disease (NAFLD) [133]. Therefore, the activity of these proteins in NAFLD has been implicated as a potential link between MIRLET7BHG and NAFLD.

### 7.2 miR-3619

The up-regulation of both CTSS and RAB11FIP4 is linked to lysosomal trafficking and autophagy. MiR-3619-5p down-regulated CTSS in THP-1-derived macrophages, leading to autophagy. miR-3619 may control cathepsin S expression, promoting autophagy in macrophages [134]. DGCR5 promotes GBC development by sponging miR-3619 and activating MEK/ERK1/2 and JNK/p38 MAPK pathways. Silencing DGCR5 induced apoptosis and altered the expression of several proteins involved in apoptosis, including BCL-2 and Bax [12].

### 7.3 Let-7a-3

Autophagy reduces the levels of SQSTM1/p62 and CALCOCO2/NDP52. SQSTM1 down-regulation inhibits DICER1 and AGO2, resulting in inhibition of migration in OVC. Increased MIRLET7A-3P promotes autophagy and inhibits OVC cell motility. Recombinant capsid protein VP1 (rVP1) of the foot-and-mouth disease virus increased BECN1-independent autophagy and reestablished SQSTM1, DICER1, and AGO2 expression, leading to the reversal of MIRLET7A-3P linked autophagy and restoration of motility. The induction of autophagy could reduce AGO2, SQSTM1, and DICER1 levels and increase MIRLET7A-3P as an efficient treatment approach for reversing OVC cell motility [135,136].

### 7.4 Let-7b

MSCs exposed to ROS can regulate autophagy and apoptosis using Let-7b. Under oxidative stress conditions, MSCs expressing Let-7b show increased expression of survival-related proteins and reduced TUNEL-positive and Annexin V/PI-stained cells. Additionally, genes associated with autophagy, such as Atg5, Atg7, Atg12, and BECN1, were significantly down-regulated in let-7b-MSCs [137].

## 8 Genetic variations in LOC124905135-derived non-coding RNAs

**Fig. 5:**
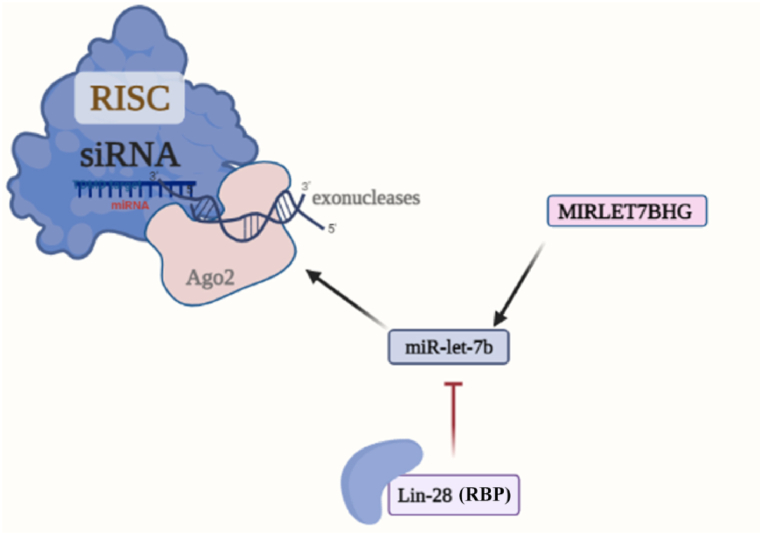
miR-let-7b maturation can be inhibited by Lin-28, a regulatory factor involved in fine-tuning microRNA biogenesis. Lin-28 is a potent RNA-binding protein, which exerts its post-transcriptional regulatory role by modulating microRNA processing, especially interfering with the maturation of miR-let-7b. Consequently, this disruption results in an increased abundance of target genes associated with cell growth, thereby implicating Lin-28 in an intricate regulatory network governing cellular proliferation and differentiation. Created with BioRender.com.

While considerable research has been conducted on genetic variants in protein-coding regions, it is also necessary to investigate the importance of non-coding variants in tumors. Some studies have revealed that somatic and germline variants occur in non-coding parts of the genome. Therefore, investigating these non-coding variants is crucial to gain a more comprehensive understanding of their role in cancer development and progression [138]. Several human tumors have been predicted to be reliant on aberrant miRNA expression. Common SNPs in miRNA genes may affect miRNA maturation or transcriptional regulation [139]. The most common mRNA stability regulators in 3′-UTRs are adenylate/uridylate-rich elements (AREs) and SNPs. Notably, miRNAs are involved in gene expression switches mediated by AREs and SNPs [140]. The let-7 microRNA cluster at chromosome 22q13.31 produces two types of let-7. The promoter of this cluster is highly responsive to NF-κB. A mutation at bp−947 has been found to reduce promoter activity. It's worth noting that the level of NF-κB responsiveness depends on the cell type. Cells with high levels of Lin-28B, an RNA-binding protein, may prevent the maturation of let-7a and let-7b [141] by binding to the RISC complex. This in turn, leads to increased production of important cancer-inducing genes such as RAS and MYC, as well as cell-cycle regulators like E2F2, CDK6, and CCND2, all of which have significant effects on tumor growth and metastasis (Fig. 5) [142].

### 8.1 MIRLET7BHG

Polymorphisms within the MIRLET7BHG gene may confer an increased susceptibility to lung cancer. Individuals carrying the polymorphic alleles rs13053856, rs11090910, rs11703832, and rs12170325, whether heterozygous or homozygous, appear to be notably vulnerable to lung cancer linked to asbestos exposure. These findings suggest a potential genetic predisposition to asbestos-induced lung carcinogenesis among individuals with specific MIRLET7BHG variants [143].

### 8.2 miR-3619

The rs619586 variant of MALAT1 may be better at sponging miR-3619-5p in GC, which was proposed to worsen the progression or enhance the chemoresistance of tumors [144].

### 8.3 miR-4763

Based on the type of cancer, XPO5 rs11077 alleles A and C have been shown to lead to a poor prognosis. The A to C transition results in a new junction for miR-4763-5p in the XPO5 mRNA, leading to a decrease in XPO5 protein expression [145]. CD151 dysregulation is observed in various cancer types and is essential for cancer progression and metastasis. Research has shown that adenylate/uridylate-rich elements (ARE) and SNPs present in CD151 can affect its interaction with transcription factors (such as ZNF519) to promote the onset of breast cancer. SNP rs1130716 was found in the hsa-miR-3663-3p binding site, and SNP rs35042031 was found in the hsa-miR-4763-3p binding site [140].

## 9 LOC124905135-derived non-coding RNAs, cancer progression, and clinical outcomes

Under physiological conditions, ncRNAs act precisely to regulate proliferation and survival pathways. However, in numerous diseases, particularly human cancer, the dysregulation of many ncRNAs has been observed. Notably, these ncRNAs have been implicated in critical tumor-promoting processes, and their aberrant expression is associated with the survival of tumor cells [146]. Certain types of ncRNAs, such as miRNAs, show altered expression patterns in malignant cells or tissues, and their expression profiles are conserved across various tissues and body fluids. Consequently, several ncRNAs have been investigated as therapeutic targets and biomarkers that are potential diagnostic/prognostic factors in cancer management [147].

### 9.1 PRR34-AS1

High expression of PRR34-AS1 was identified as a cancer risk factor, and up-regulation of this ncRNA is associated with poor prognosis, especially in HCC. The PRR34-AS1 gene has been linked to the progression of HCC [148]. In AML patients, higher PRR34-AS1 expression was correlated with worse OS and fewer complete remissions after chemotherapy compared to those with low PRR34-AS1 expression levels [98].

### 9.2 MIRLET7BHG

Pyroptosis is programmed cell death facilitated by gasdermins (GSDMs). In a study of glioma patients, researchers investigated whether MIRLET7BHG regulates glioma progression by up-regulating GSDMs. The downstream target of MIRLET7BHG was identified, and the ceRNA network was demonstrated. MIRLET7BHG was negatively correlated with tumor stage and patient survival. As the cancer stage progresses, the survival rates become shorter, and the level of MIRLET7BHG expression is increased. They concluded that high MIRLET7BHG levels were correlated with a better prognosis in glioma patients [149].

### 9.3 miR-3619

Interestingly, Tan and coworkers found that circ-ZFR knockdown meaningfully inhibited cell proliferation and EMT in HCC by boosting the expression of CTNNB1 through miR-3619-5p sponge. Furthermore, microarray and Gene Ontology (GO) analysis revealed that circ-ZFR might affect the Wnt/β-catenin pathway. Overall, Circ-ZFR may contribute to HCC by regulating the miR-3619-5p/CTNNB1 axis and promoting the Wnt/β-catenin pathway [74]. miR-3619-3p was found to act as an oncogene that targets Wnt/β-catenin pathway proteins in PTC (papillary thyroid cancer) cell lines, thus enhancing migration and invasion. They also investigated the association between the expression of miR-3619-3p and PTC patient's clinicopathological status. They used the scratch wound repair method to evaluate the function of miR-3619-3p in PTC. miR-3619-3p was up-regulated in PTC cells and initiated the Wnt/β-catenin pathway by maintaining the β-catenin mRNA stability, thereby increasing migration and invasion [150].

In an investigation, Si and colleagues conducted experiments to explore the HEIH role in OVC. They reported that HEIH was highly expressed in ovarian tumor tissues and cells. High expression of HEIH correlates with poor prognosis, with a survival rate of only 25 % and increased rates of PMI, while inhibiting cellular senescence in OVC. It was proposed that HEIH acts as a sponge for miR-3619-5p and mediates the regulation of OVC. The up-regulation of cortactin-binding protein 2 (CTTNBP2), a downstream target of miR-3619-5p, abrogated the suppressive effects of HEIH knockdown on OVC. Alternatively, via regulating CTTNBP2 expression, HEIH could promote cell PMI while reducing cell senescence in OVC by modulating the miR-3619-5p/CTTNBP2 axis [151].

In another interesting study, Sun et al. measured the expression levels of LINC00958 and miR36195p, as well as their potential mechanisms of action in CRC. Transfection of short hairpin RNA (shRNA)-LINC00958 into cells led to up-regulation of LINC00958 expression, while miR-3619-5p was down-regulated in CRC cells. ShRNA-LINC00958 inhibited proliferation, invasion, and migration while boosting apoptosis in CRC cells by increasing the expression of Bax and caspase-3 while suppressing Bcl-2. Using a luciferase reporter assay with a miR36195p inhibitor, they showed that LINC00958 could specifically target miR36195p, thereby affecting cell growth, invasion, migration, and PCD [152].

Although the lncRNA DGCR5 (DiGeorge syndrome critical region) is associated with various tumors, its role in gallbladder cancer (GBC) is not well understood. Compared to normal counterparts, DGCR5 was found to be higher in GBC tissue samples and cell lines. Likewise, they suggested that DGCR5 carries out functions, including promoting PMI by targeting the miR-3619-5p. Transfection with miR-3619-5p and suppression of DGCR5 both significantly reduced cell PMI, and inactivated the MEK/ERK1/2 and JNK/p38 MAPK pathways, and overexpression of DGCR5 demonstrated these potential effects on GBC tumor through these pathways [12]. The expression levels of miR-3619 and its effects on different cancer types are summarized in Table 1.

**Table 1:** The expression levels of miR-3619 and its effects on different cancer types.

Cancer	miR-3619 expression	Inhibitor CeRNA	Target	Mechanism	Result	Sample	Methodology	Ref.
Prostate cancer	Down-regulated	N.A.	CDKN1C promoter/CDK4, 6 and cyclin-D1	Reduced expression of CDKN1C, overexpression of CDK4, 6 and cyclin-D1	Cell growth	Prostate cancer DU145 and PC3 cell lines	*In vitro* cell transfection, qRT-PCR, Western blotting	[153]
Hepatocellular carcinoma (HCC)	Down-regulated	Circ-ZFR	CTNNB1	Activated Wnt/β-catenin pathway	Proliferation & EMT	HCC tissues	qRT-PCR	[74]
	Down-regulated	LncRNA -PVT1	MKL1	MKL1 (a transcriptional coactivator) was overexpressed by PVT1 through miR-3619-5p sponging	Cell migration & invasion	HCC cell lines (HepG2 & Cos-7c)	qRT-PCR, ChIP-assay, *in vitro* cell transfection	[84]
	Down-regulated	CircCMTM3	SOX9	Knockdown of circCMTM3 suppressed angiogenesis and HCC tumor growth	Angiogenesis, migration, invasion	HCC tissue, umbilical vein endothelial cells (HUVECs), HCC cell lines (Huh7, Hep3B, HCCLM3, SK‐HEP‐1, & MHCC97)	qRT-PCR, Western blotting, *in vitro* cell transfection	[154]
Retinoblastoma (RB)	Down-regulated	LINC00202	RIN1	Overexpression of RIN1 by suppressing miR-3619	Poor prognosis, cell proliferation, migration & invasion	Weri-Rb1 cells & tissue	qRT-PCR, *in vitro* cell transfection	[76]
	Down-regulated	LncRNA -HCP5	HDAC9	LncRNA -HCP5 targeted miR-3619 to enhance HDAC9 overexpression	Cell viability, migration, & invasion	RB tissue & cell lines (Y79, HXO-RB44, WERI-Rb-1, & SO-RB50), mouse tumors	qRT-PCR, mouse tumors (*in vivo*), *in vitro* cell transfection	[100]
	Down-regulated	LncRNA - NEAT1	LASP1	LncRNA-NEAT1 targeted miR-3619-5p & enhanced LASP1 expression	Migration, invasion, & proliferation; apoptosis & cell cycle arrest	RB tissue & cell lines (Y-79 and SO-RB50)	qRT-PCR, *in vitro* cell transfection, Western blotting	[155]
Colorectal cancer (CRC)	Down-regulated	LINC00958	N.A.	Enhanced Bcl-2; decreased the expression of Bax & caspase-3	Reduced apoptosis, proliferation, invasion, migration	CRE (SW480) cells	qRT-PCR, *in vitro* cell transfection, and Western blot	[152]
	Down-regulated	LINC00882	pcDNA3.1/CTNNB1	Silencing miR-3619 led to EMT by increasing LINC00882 & pcDNA3.1/CTNNB1	EMT & invasion	CRC tissues & cell lines	qRT-PCR, cell invasion assay (*in vitro*), and Western blot	[156]
Osteosarcoma	Down-regulated	LINC00665	N.A.	Sponging miR-3619 through LINC00665 promoted proliferation & progression	Proliferation & invasion	Tissue & OS cell lines (143B, U2OS, MG63, Saos-2)	qRT-qPCR, *in vitro* cell transfection	[157]
Papillary thyroid carcinoma (PTC)	Up-regulated	N.A.	N.A.	Activated Wnt/β-catenin by increasing the stability of β-catenin mRNA	Migration & invasion	PTC tissue & cell lines (BCPAP, K1, TPC-1)	qRT-PCR, *in vitro* cell transfection, and Western blot	[150]
Breast cancer	Down-regulated	LINC00665	β-catenin	Enhanced β-catenin expression by targeting miR-3619-5p	PMI, inhibited apoptosis	BC tissue & cell lines (MDA-MB-231, MCF-7)	qRT-PCR, *in vitro* cell transfection, and Western blot	[158]
Gallbladder cancer	Down-regulated	DGCR5	N.A.	Activated MEK/ERK1/2 & JNK/p38 MAPK pathways	PMI	GBC tissue & cell lines (NOZ, SGC-996, GBC-SD, OCUG, and 293T)	qRT-PCR, *in vitro* cell transfection, and Western blot	[12]
Ovarian cancer	Down-regulated	HEIH	CTTNBP2	HEIH targeted miR-3619-5p which directly targeted CTTNBP2	PMI, inhibited cell senescence	OVC tissue & cell lines (OVCA429, OVCA433, OVCAR3)	qRT-PCR, *in vitro* cell transfection, and Western blot	[151]
Non-small cell lung cancer	Down-regulated	HOXA11-AS	SALL4	HOXA11-AS targeted miR-3619 to promote SALL4 overexpression	PMI, glycolysis, promoted apoptosis	NSCLC tissue & cell lines (H1299, A549), mouse tumors	qRT-PCR, xenograft model (*in vivo*), *in vitro* cell transfection, and Western blot	[159]
	Down-regulated	Circ_0026134	CHAF1B	Circ_0026134 directly targeted miR-3619-5p; circ_0026134 regulated CHAF1B	Colony formation, migration, invasion, glycolysis, promoted apoptosis	NSCLC tissue & cells (H520, A549)	qRT-PCR, *in vitro* cell transfection, and Western blot	[13]
Stomach adenocarcinoma	Down-regulated	STAT4	TBC1D10B	STAT4-repressed miR-3619-5p by modifying the downstream TBC1D10B target gene	PMI, tumor growth	STAD tissue & cell lines (MGC-803, HGC-27, SGC-7901, BGC-823, AGS)	qRT-PCR, ChIP-assay, *in vitro* cell transfection, and Western blot	[82]
Bladder cancer (BC)	Down-regulated	N.A.	β-catenin, CDK2, & p21 gene promoter	Directly targeted β-catenin & CDK2; activated p21 gene promoters	Proliferation, migration, & invasion	BC tissue & cell lines (5637, EJ, T24, J82), mouse tumors	qRT-PCR, mouse tumor (*in vivo*), and *in vitro* cell transfection	[160]

### 9.4 miR-4763

Next-generation deep sequencing was employed by Wu et al. to measure the circ-RNAs profiles in bone marrow samples that the expression of hsa_circ_0012152 could discriminate AML-M5 patients from normal donors. They claimed Consistent with their predictions, the interaction of hsa_circ_0012152 with hsa-miR-4763-3p and its related mRNAs could promote invasion in AML-M5 [161]. The overexpression of hsa_circRNA_000121 in PTMC and its linkage with papillary thyroid microcarcinoma (PTMC) invasiveness has been documented. An evaluation of the gene expression using qRT-PCR in metastatic and non-metastatic PTMC tissue specimens showed that the expression of hsa_circRNA_000121, SRC, and MMP-14 was higher in the non-metastatic group, whereas the hsa-miR-4763 expression was lower. In addition, the non-metastatic group had higher levels of hsa_circ_000121 expression in the peripheral blood compared to the metastatic group. They concluded that hsa_circRNA_000121 could up-regulate SRC expression by sponging hsa-miR-4763, thus increasing the invasiveness of PTMC, ultimately giving rise to cervical lymph node metastasis [88].

### 9.5 Let-7b

Zebularine (a nucleoside analog of cytidine) was used to inhibit the invasion ability of three human CRC cell lines, oxaliplatin-resistant SW620 (SW620/OxR), SW620, and SW480. Zebularine up-regulated the intracellular expression of let-7b in SW620 and SW620/OxR cells, indicating that it may inhibit aggressiveness in highly aggressive CRC cells by up-regulating the intracellular expression of let-7b. The DNA methylation status upstream of the let-7b coding region (CpG island near TSS of MIRLET7BHG7) was not altered by the treatment with zebularine [162]. When the transcription level of mir-let7b-3p was investigated, it appeared to regulate the PTEN expression in prostate cancer tissue samples in comparison with benign prostate hyperplasia (BPH) and normal adjacent tissue samples. The miR-let7b with down-regulation and the mir-548 with up-regulation expression were associated with high-risk Gleason scores. They suggested that miR-let7b may be a new target for prostate cancer treatment [163].

## 10 Conclusions and future perspective

The LOC124905135 gene encodes a variety of ncRNAs, including miR-4763, miR-3619, as well as MIRLET7B, PRR34-AS1, and MIRLET7BHG, which serve as the host genes for miR-let7b, miR-let7-a3, and miR-4763 respectively. These three miRNAs are believed to be transcribed from the 3′UTR of MIRLET7BHG. Furthermore, the LOC112268288 ncRNA appears to originate from the first intron of MIRLET7BHG (or alternatively the X1 variant of LOC124905135), which is associated with the tumor suppressor miR-3619. PRR34-AS1 is another ncRNA that appears to arise from the first exon of the LOC124905135-X3 variant.

A comprehensive understanding of the complex roles of non-coding RNAs, including those derived from LOC124905135, in cellular functions and disease pathogenesis is crucial. Elucidating their molecular functions can lead to new opportunities for targeted therapies and precision medicine approaches. Conducting additional research in this area will improve our understanding of cancer biology, and may lead to innovative treatments to improve patient outcomes. As a result, abnormalities in gene expression of the ncRNAs in this cluster have been reported in various cancer types. Furthermore, the activity of these ncRNAs has been implicated in several cancer types and is thought to be critical for cancer development. To promote the future use of ncRNAs in medicine, it is necessary to conduct thorough and comprehensive research into the relationship between the expression and function of these RNAs in various biological systems, and medical diseases, particularly those in oncology and immunology. Understanding the tissue-specific transcriptional profiles of lncRNAs is critical for the development of biomarkers for cancer diagnosis and prognostic or prospective targets. Focusing on clusters of ncRNAs for therapeutic targeting may be more productive than individual ncRNAs.

The first step is usually large-scale functional genomic research. The computational resources required to investigate lncRNAs are beginning to become available to reveal the lncRNAs role in malignancies. Current integrated online platforms possess common characteristics and share similar features, which might result in shortening the time needed for scientists to achieve their goals. At present the bioinformatics tools for functional evaluation of lncRNAs, include LncExpDB, LNCipedia, lncRNome, LncBook, NONCODE, LncSEA, LncRNASNP, LncRNA2Target, LncLocator, LncRNADisease, LncTarD, AnnoLnc, TANRIC, Lnc2Catlas, Lnc2Cancer, LnCAR, and ncRNA-eQTL, and LncR2Metasta.

The use of miRNA therapy still faces challenges such as toxicity, adverse effects, and low efficacy at high doses that need to be addressed. Fortunately, advancements in biochemistry and bioengineering technologies have made it possible to tackle these obstacles in miRNA therapy. Recent preclinical and clinical trials have demonstrated improved safety, efficacy, and specificity of new synthetic RNA oligomers for miRNA mimics and antisense miRNA inhibitors (mimics and antagonists). Given the emerging strategy of personalized cancer medicine that focuses on discovering prospective biomarkers and new targets for treating and diagnosing various cancers, miRNAs may be a practical option due to their established role, tissue specificity, and evolutionary conservation. Furthermore, promising new delivery systems have been created and utilized to ensure the efficient and precise delivery of miRNA drugs. With the notable progress of miRNA therapy, miRNA therapy will likely emerge as a leading next-generation cancer therapy through the application of advanced synthetic RNA technologies and cancer-specific delivery systems [54,164,165].

Various tools are available to identify and evaluate the performance of miRNAs, including miRMaster, miEAA, mistargeting, miRSwitch, and novoMiRank, which provide a comprehensive analysis of all the features of miRNAs. Data sources for miRNAs and their variants, such as miRCarta, miRSNPdb, miRPathDB, and miRATbase, are also available. TissueAtlas, CellTypeAtlas, and ATmiRes are databases that cover miRNA transcription patterns in the transcriptome. In addition, current miRNA software systems offer services such as converting miRNA nomenclature or constructing reporter assays, including miRTaH, miRBaseConverter, and miBlast [166]. Some basic information about miR-3619, miR-let7-A3, miR-let-7b, and miR-4763, obtained using the miRNASNP-v3 web server available at http://bioinfo.life.hust.edu.cn/miRNASNP#!/is presented in Table 2.

**Table 2:** Basic information related to four miRNAs that are derived from LOC124905135.

miRNA	Genome position^a^	Host gene	Mature miRNA
hsa-miR-3619	chr22:46,091,044–46,091,126	Intergenic	hsa-miR-3619-5p
			hsa-miR-3619-3p
hsa-let-7a-3	chr22:46,112,749–46,112,822	Intergenic	hsa-let-7a-5p
			hsa-let-7a-3p
hsa-let-7b	chr22:46,113,686–46,113,768	Intergenic	hsa-let-7b-5p
			hsa-let-7b-3p
hsa-miR-4763	chr22:46,113,566–46,113,657	Intergenic	hsa-miR-4763-5p
			hsa-miR-4763-3p

a Genome Position gathered from NCBI database based on Annotation release RS_2023_03, Assembly GRCh38.p14 (GCF_000001405.40).

Despite significant advances in human gene research, there is still a knowledge gap regarding the effect of SNPs and miRNAs and the corresponding regulatory mechanisms involved. Additional research is needed to unravel the complex interplay between these two genetic elements and their potential implications for human health and disease. According to research, SNPs in the miRNA-mRNA interaction motif may affect the functional role of miRNAs. Many recent studies have shown that the presence of SNPs in the functional roles of miRNAs that interact with the 3′UTR of the target gene, i.e., the seed region, reduces the microRNA function. Due to the relatively limited experimental study on the influence of SNPs on the expression and function of miR-3619, miR-let7-A3, miR-let-7b, and miR-4763 SNPs in cancer, we collected these SNPs from miRNASNP-v3 (Table 3) [167].

**Table 3:** Information gathered from miRNASNPv3 displaying the frequency of microRNA mutations as well as their location, allele, location, and enthalpy.

Pre-miRNA	Mutation ID	Position	Ref/Alt	Cancer Type	Region	Mature miRNA	ΔG^a^	Express
Let-7a-3	COSN29468566	chr22:46112788	G/T	Liver	pre-miRNA	N.A.	−0.5	Up
Let-7b	COSN26568819	chr22:46113729	G/T	Liver	pre-miRNA	N.A.	5	Down
	COSN15618723	chr22:46113730	C/CCT	Oesophagus; carcinoma	pre-miRNA	N.A.	2.2	Down
	COSN28891480	chr22:46113760	C/T	Large intestine; carcinoma	Mature	Let-7b-3p	4.6	Down
	COSN20080146	chr22:46113767	T/C	Prostate; carcinoma	pre-miRNA	N.A.	−2.2	Up
	COSN28758378	chr22:46113768	G/C	Lung; carcinoma	pre-miRNA	N.A.	1	Up
mir-4763	COSN6563154	chr22:46113652	G/A	Central nervous system; primitive neuroectodermal tumour-medulloblastoma	pre-miRNA	N.A.	0.71	Up
mir-3619	COSN23853617	chr22:46091069	G/C	Upper aerodigestive tract; carcinoma	Mature	miR-3619-5p	5.1	Down

a ΔG: The variation in MFE between the wild type and mutant alleles.

It is clear that some LOC124905135-derived ncRNAs play a role in cancer. However, their function is not yet fully understood due to complex functional pathways and limitations in methodology. In-depth research could improve clinical diagnosis, including imaging and various molecular approaches to enhance diagnostic sensitivity. A comprehensive understanding of the transcription levels and activity of regulatory RNAs, and characterization of ncRNA control pathways could greatly improve our knowledge. One approach to understanding the function of ncRNAs in cells is to examine them without manipulating their associated genes. Among the various ncRNAs, the action of LOC124905135-derived ncRNAs in cancer has been primarily focused on the suppression or inhibition of miRNAs, with little consideration of other possible functions. A thorough understanding of LOC124905135-derived ncRNAs will likely result in a multitude of downstream effectors, as opposed to a single one, making future treatments more practical. Therefore, further research is needed to explore this gene region in order to reveal therapeutic targets for cancer treatment.

## CRediT authorship contribution statement

**Maryam Eftekhari Kenzerki:** Writing – review & editing, Writing – original draft, Visualization, Validation, Investigation, Data curation, Conceptualization. **Amirhossein Mohajeri Khorasani:** Writing – review & editing, Writing – original draft, Validation, Investigation, Data curation. **Iman Zare:** Writing – review & editing, Writing – original draft, Visualization, Validation, Investigation, Data curation. **Farzane Amirmahani:** Visualization, Validation, Investigation, Data curation. **Younes Ghasemi:** Writing – review & editing, Validation, Investigation, Data curation. **Michael R. Hamblin:** Writing – review & editing, Validation, Investigation. **Pegah Mousavi:** Writing – review & editing, Writing – original draft, Visualization, Validation, Supervision, Project administration, Investigation, Data curation, Conceptualization.

## Ethics approval and consent to participate

Not applicable.

## Data availability statement

The data associated with this study are drawn from publicly available databases. However, the data used in the current study are available from the corresponding author upon reasonable request.

## Funding

Not applicable.

## Declaration of competing interest

The authors declare that they have no known competing financial interests or personal relationships that could have appeared to influence the work reported in this paper.

## Abbreviations

ALL: Acute lymphoblastic leukemia
AML: Acute myeloid leukemia
APL: Acute promyelocytic leukemia
AREs: Adenylate/uridylate-rich elements
ACAN: Aggrecan
BPH: Benign prostate hyperplasia
BC: Bladder cancer
BMP2: Bone morphogenetic protein 2
CPT1: Carnitine palmitoyl-transferase 1
CTSS: Cathepsin S
ChIP: Chromatin immunoprecipitation
circRNAs: Circular RNAs
COL2A1: Collagen type II α 1 chain
COL3A1: Collagen type III α1
CR: Complete remission
CTTNBP2: Cortactin-binding protein 2
CRC: Colorectal cancer
CeRNA: Competing endogenous RNA
CLIP: Cross-linking and immunoprecipitation
cSCC: Cutaneous squamous cell carcinoma
CDK: Cyclin-dependent kinase
DGCR5: DiGeorge syndrome critical region
E2F2: E2F transcription factor 2
ETMR: Embryonal tumor with multilayered rosettes
EZH2: Enhancer of zester homolog-2
EMT: Epithelial-mesenchymal transition
ECM: Extracellular matrix
FAO: Fatty acid oxidation
FISH: Fluorescence in situ hybridization
FOXO3: Ffork-head box O3
GBC: Gallbladder cancer
GSDMs: Gasdermins
GC: Gastric cancer
GO: Gene ontology
GDC: Genomic data commons
GTEx: Genotype-tissue expression
HDGF: Heavily germanium-doped fiber
Hh: Hedgehog
HCC: Hepatocellular carcinoma
HCP5: Histocompatibility leukocyte antigen complex P5
HDGF: Hepatoma-derived growth factor
HDAC9: Histone deacetylase C9
hBMSC: Human bone-marrow stem cell
HEIH: Highly expressed in hepatocellular carcinoma
HGDF: Heavily Germanium-Doped Fiber
IGF1R: Insulin growth factor 1 receptor
KPNA4: Karyopherin subunit alpha 4
lincRNA: Long intergenic non-coding RNA
LINC02939: Long intergenic non-coding RNA 2939
lncRNAs: Long non-coding RNAs
MSCs: Mesenchymal stem cells
mRNAs: Messenger RNAs
MSP: Methylation-specific PCR
miRNAs: MicroRNAs
MFE: Minimum free energy
MLL: Mixed-lineage leukemia
NATs: Natural antisense transcripts
NAFLD: Nonalcoholic fatty liver disease
ncRNAs: Non-coding RNAs
NEAT1: Nuclear-enriched abundant transcript 1
NSCLC: Non-small cell lung cancer
OSCC: Oral squamous cell carcinoma
OS: Overall survival
PAAD: Pancreatic adenocarcinoma
PDAC: Pancreatic ductal adenocarcinomas
PTC: Papillary thyroid cancer
PTMC: Papillary thyroid microcarcinoma
PPARG: Peroxisome proliferator-activated receptor gamma
PPARGC1A: Peroxisome proliferator-activated receptor gamma coactivator 1 alpha
PGC1α: PPAR coactivator-1α
PCD: Programmed cell death
PRR34-AS1: PRR34 antisense RNA 1
RAB11FIP4Rab11: family-interacting protein 4
RFA: Radiofrequency ablation
RRTS: Radix ranunculus temate saponins
RB: Retinoblastoma
RBP: RNA-binding protein
RIP: RNA immunoprecipitation
shRNA: Short hairpin RNA
SIRT1: Silent information regulator 1
SNPs: Single nucleotide polymorphisms
SNVs: Single nucleotide variants
siRNAs: Small interfering RNAs
SOX12: SRY-box transcription factor 12
STAD: Stomach adenocarcinoma
TCGA: The cancer genome atlas
TF: Transcription factor
TNBC: Triple-negative breast cancer
TNF-α: Tumor necrosis factor α

## References

[1] (2020). Pan-cancer analysis of whole genomes. Nature.

[2] Chen J., Mai H., Chen H., Zhou B., Hou J., Jiang D.K. (2021). Pan-cancer analysis identified C1ORF112 as a potential biomarker for multiple tumor types. Front. Mol. Biosci..

[3] Hanahan D. (2022). Hallmarks of cancer: new dimensions. Cancer Discov..

[4] Anastasiadou E., Jacob L.S., Slack F.J. (2018). Non-coding RNA networks in cancer. Nat. Rev. Cancer.

[5] Hausser J., Mayo A., Keren L., Alon U. (2019). Central dogma rates and the trade-off between precision and economy in gene expression. Nat. Commun..

[6] Yin Y., Yan P., Lu J., Song G., Zhu Y., Li Z. (2015). Opposing roles for the lncRNA haunt and its genomic locus in regulating HOXA gene activation during embryonic stem cell differentiation. Cell Stem Cell.

[7] Jain A.K., Xi Y., McCarthy R., Allton K., Akdemir K.C., Patel L.R. (2016). LncPRESS1 is a p53-regulated LncRNA that safeguards pluripotency by disrupting SIRT6-mediated de-acetylation of histone H3K56. Mol. Cell.

[8] Amirmahani F., Vallian S., Asadi M.H. (2023). The LncRNA MIAT is identified as a regulator of stemness-associated transcript in glioma. Mol. Biol. Rep..

[9] Kumarswamy R., Volkmann I., Thum T. (2011). Regulation and function of miRNA-21 in health and disease. RNA Biol..

[10] Statello L., Guo C.-J., Chen L.-L., Huarte M. (2021). Gene regulation by long non-coding RNAs and its biological functions. Nat. Rev. Mol. Cell Biol..

[11] Gil N., Ulitsky I. (2020). Regulation of gene expression by cis-acting long non-coding RNAs. Nat. Rev. Genet..

[12] Liu S., Chu B., Cai C., Wu X., Yao W., Wu Z. (2020). DGCR5 promotes gallbladder cancer by sponging MiR-3619-5p via MEK/ERK1/2 and JNK/p38 MAPK pathways. J. Cancer.

[13] Ge L., Tan W., Li G., Gong N., Zhou L. (2022). Circ_0026134 promotes NSCLC progression by the miR-3619-5p/CHAF1B axis. Thoracic Cancer.

[14] Huang L., Wang L., Wang L., Wu Z., Jiang F., Li Q. (2021). MicroRNA let-7b inhibits proliferation and induces apoptosis of castration-resistant prostate cancer cells by blocking the Ras/rho signaling pathway via NRAS. CTS-CLINICAL AND TRANSLATIONAL SCIENCE.

[15] Wang Y., Zhang B., Zhu Y., Zhang Y., Li L., Shen T. (2021).

[16] Li H., Zhang X., Wang F., Zhou L., Yin Z., Fan J. (2016). MicroRNA-21 lowers blood pressure in spontaneous hypertensive rats by upregulating mitochondrial translation. Circulation.

[17] Fatehi Z., Amirmahani F., Tavassoli M. (2019). Association study of TAAAA polymorphism in the first intron of p53 gene with risk of colorectal cancer in Iranian population. Egyptian Journal of Medical Human Genetics.

[18] Bartel D.P. (2004). MicroRNAs: genomics, biogenesis, mechanism, and function. cell.

[19] Yoshida K., Yokoi A., Yamamoto Y., Kajiyama H. (2021). ChrXq27.3 miRNA cluster functions in cancer development. J. Exp. Clin. Cancer Res..

[20] Wei R., Yang J., Liu G-q, Gao M-j, Hou W-f, Zhang L. (2013). Dynamic expression of microRNAs during the differentiation of human embryonic stem cells into insulin-producing cells. Gene.

[21] Ono K., Horie T., Nishino T., Baba O., Kuwabara Y., Yokode M. (2015). MicroRNA-33a/b in lipid metabolism–novel “thrifty” models. Circ. J..

[22] Kabekkodu S.P., Shukla V., Varghese V.K., D' Souza J., Chakrabarty S., Satyamoorthy K. (2018). Clustered miRNAs and their role in biological functions and diseases. Biol. Rev..

[23] Calin G.A., Sevignani C., Dumitru C.D., Hyslop T., Noch E., Yendamuri S. (2004). Human microRNA genes are frequently located at fragile sites and genomic regions involved in cancers. Proc. Natl. Acad. Sci. USA.

[24] Farazi T.A., Hoell J.I., Morozov P., Tuschl T. (2013). MicroRNA Cancer Regulation.

[25] Oliayi A.J., Asadi M.H., Amirmahani F. (2019). SNHG6 203 transcript could be applied as an auxiliary factor for more precise staging of breast cancer. J. Kerman Univ. Med. Sci..

[26] Morris K.V., Mattick J.S. (2014). The rise of regulatory RNA. Nat. Rev. Genet..

[27] Ulitsky I., Bartel David P. (2013). lincRNAs: genomics, evolution, and mechanisms. Cell.

[28] Derrien T., Johnson R., Bussotti G., Tanzer A., Djebali S., Tilgner H. (2012). The GENCODE v7 catalog of human long noncoding RNAs: analysis of their gene structure, evolution, and expression. Genome Res..

[29] Bridges M.C., Daulagala A.C., Kourtidis A. (2021). LNCcation: lncRNA localization and function. J. Cell Biol..

[30] Chen R.-X., Chen X., Xia L.-P., Zhang J.-X., Pan Z.-Z., Ma X.-D. (2019). N6-methyladenosine modification of circNSUN2 facilitates cytoplasmic export and stabilizes HMGA2 to promote colorectal liver metastasis. Nat. Commun..

[31] Zhang J., Zhang X., Li C., Yue L., Ding N., Riordan T. (2019). Circular RNA profiling provides insights into their subcellular distribution and molecular characteristics in HepG2 cells. RNA Biol..

[32] Mukherjee N., Calviello L., Hirsekorn A., de Pretis S., Pelizzola M., Ohler U. (2017). Integrative classification of human coding and noncoding genes through RNA metabolism profiles. Nat. Struct. Mol. Biol..

[33] Cabili M.N., Dunagin M.C., McClanahan P.D., Biaesch A., Padovan-Merhar O., Regev A. (2015). Localization and abundance analysis of human lncRNAs at single-cell and single-molecule resolution. Genome Biol..

[34] Zuckerman B., Ron M., Mikl M., Segal E., Ulitsky I. (2020). Gene architecture and sequence composition underpin selective dependency of nuclear export of long RNAs on NXF1 and the TREX complex. Mol. Cell.

[35] Zhang X., Wang W., Zhu W., Dong J., Cheng Y., Yin Z. (2019). Mechanisms and functions of long non-coding RNAs at multiple regulatory levels. Int. J. Mol. Sci..

[36] Grote P., Wittler L., Hendrix D., Koch F., Währisch S., Beisaw A. (2013). The tissue-specific lncRNA Fendrr is an essential regulator of heart and body wall development in the mouse. Dev. Cell.

[37] Mondal T., Subhash S., Vaid R., Enroth S., Uday S., Reinius B. (2015). MEG3 long noncoding RNA regulates the TGF-β pathway genes through formation of RNA–DNA triplex structures. Nat. Commun..

[38] Tsai M.-C., Manor O., Wan Y., Mosammaparast N., Wang J.K., Lan F. (2010). Long noncoding RNA as modular scaffold of histone modification complexes. Science.

[39] Mondal T., Rasmussen M., Pandey G.K., Isaksson A., Kanduri C. (2010). Characterization of the RNA content of chromatin. Genome Res..

[40] Du Z., Sun T., Hacisuleyman E., Fei T., Wang X., Brown M. (2016). Integrative analyses reveal a long noncoding RNA-mediated sponge regulatory network in prostate cancer. Nat. Commun..

[41] Grelet S., Link L.A., Howley B., Obellianne C., Palanisamy V., Gangaraju V.K. (2017). A regulated PNUTS mRNA to lncRNA splice switch mediates EMT and tumour progression. Nat. Cell Biol..

[42] Lin A., Li C., Xing Z., Hu Q., Liang K., Han L. (2016). The LINK-A lncRNA activates normoxic HIF1α signalling in triple-negative breast cancer. Nat. Cell Biol..

[43] Rackham O., Mercer T.R., Filipovska A. (2012). The human mitochondrial transcriptome and the RNA-binding proteins that regulate its expression. WIREs RNA.

[44] Vendramin R., Verheyden Y., Ishikawa H., Goedert L., Nicolas E., Saraf K. (2018). SAMMSON fosters cancer cell fitness by concertedly enhancing mitochondrial and cytosolic translation. Nat. Struct. Mol. Biol..

[45] Yang M., Lu H., Liu J., Wu S., Kim P., Zhou X. (2022). lncRNAfunc: a knowledgebase of lncRNA function in human cancer. Nucleic Acids Res..

[46] Lorenz R., Bernhart S.H., Höner Zu Siederdissen C., Tafer H., Flamm C., Stadler P.F. (2011). ViennaRNA package 2.0. Algorithms Mol Biol.

[47] Hofacker I.L. (2003). Vienna RNA secondary structure server. Nucleic Acids Res..

[48] Wang E, Lenferink A, O’Connor-McCourt M (2007 Jul). Cancer systems biology: exploring cancer-associated genes on cellular networks. Cell. Mol. Life Sci..

[49] Liu Z., Li Z., Xu B., Yao H., Qi S., Tai J. (2020). Long noncoding RNA PRR34-AS1 aggravates the progression of hepatocellular carcinoma by adsorbing microRNA-498 and thereby upregulating FOXO3. Cancer Manag. Res..

[50] Sheng J., Lv E., Xia L., Huang W. (2022). Emerging roles and potential clinical applications of long non-coding RNAs in hepatocellular carcinoma. Biomed. Pharmacother..

[51] Yang X., Song D., Zhang J., Feng H., Guo J. (2021). PRR34-AS1 sponges miR-498 to facilitate TOMM20 and ITGA6 mediated tumor progression in HCC. Exp. Mol. Pathol..

[52] Qin M., Meng Y., Luo C., He S., Qin F., Yin Y. (2021). lncRNA PRR34-AS1 promotes HCC development via modulating Wnt/β-catenin pathway by absorbing miR-296-5p and upregulating E2F2 and SOX12. Mol. Ther. Nucleic Acids.

[53] Zhao C., Wang Y., Tu F., Zhao S., Ye X., Liu J. (2021). A prognostic autophagy-related long non-coding RNA (ARlncRNA) signature in acute myeloid leukemia (AML). Front. Genet..

[54] Chen S., Li X., Zhang J., Li L., Wang X., Zhu Y. (2021). Six mutator-derived lncRNA signature of genome instability for predicting the clinical outcome of colon cancer. J. Gastrointest. Oncol..

[55] Kutilin D.S., Gusareva M.A., Kosheleva N.G., Zinkovich M.S., Gvaramiya A.K., Gappoeva M. (2021).

[56] Wu L., Li X., Liu J., Wu D., Wu D., Xu P. (2021). The Evidence from Bioinformatic Analysis.

[57] Wu L., Zheng Y., Ruan X., Wu D., Xu P., Liu J. (2022). Long-chain noncoding ribonucleic acids affect the survival and prognosis of patients with esophageal adenocarcinoma through the autophagy pathway: construction of a prognostic model. Anti Cancer Drugs.

[58] Zhang F., Jiang H., Wang N., Xu S., Zhang Y. (2021). Comprehensive network analysis of different subtypes of molecular disorders in lung cancer. American Journal of Translational Research.

[59] Hirvonen A.P. (2020).

[60] Liu L, Liao R, Wu Z, Du C, You Y, Que K, Duan Y, Yin K, Ye W (2022 Sep 17). Hepatic stellate cell exosome-derived circWDR25 promotes the progression of hepatocellular carcinoma via the miRNA-4474-3P-ALOX-15 and EMT axes. Biosci Trends.

[61] Xia Y., Zhen L., Li H., Wang S., Chen S., Wang C. (2021). MIRLET7BHG promotes hepatocellular carcinoma progression by activating hepatic stellate cells through exosomal SMO to trigger Hedgehog pathway. Cell Death Dis..

[62] Lambo S., Gröbner S.N., Rausch T., Waszak S.M., Schmidt C., Gorthi A. (2019). The molecular landscape of ETMR at diagnosis and relapse. Nature.

[63] Lambo S., von Hoff K., Korshunov A., Pfister S.M., Kool M. (2020). ETMR: a tumor entity in its infancy. Acta Neuropathol..

[64] Tuo Z., Zi H., He Q., Meng J., Hu Y., Li Y. (2021).

[65] Glaß M., Dorn A., Hüttelmaier S., Haemmerle M., Gutschner T. (2020). Comprehensive analysis of LincRNAs in classical and basal-like subtypes of pancreatic cancer. Cancers.

[66] Ooki A., Del Carmen Rodriguez Pena M., Marchionni L., Dinalankara W., Begum A., Hahn N.M. (2018). YAP1 and COX2 coordinately regulate urothelial cancer stem-like CellsYAP1 and COX2 in bladder cancer. Cancer Res..

[67] Kozar I., Philippidou D., Margue C., Gay L.A., Renne R., Kreis S. (2021). Cross-linking ligation and sequencing of hybrids (qCLASH) reveals an unpredicted miRNA Targetome in melanoma cells. Cancers.

[68] Wichmann I.A. (2020).

[69] Hafner M., Katsantoni M., Köster T., Marks J., Mukherjee J., Staiger D. (2021). CLIP and complementary methods. Nature Reviews Methods Primers.

[70] Winter J., Jung S., Keller S., Gregory R.I., Diederichs S. (2009). Many roads to maturity: microRNA biogenesis pathways and their regulation. Nat. Cell Biol..

[71] Casey M.C., Prakash A., Holian E., McGuire A., Kalinina O., Shalaby A. (2019). Quantifying Argonaute 2 (Ago2) expression to stratify breast cancer. BMC Cancer.

[72] Piskounova E., Polytarchou C., Thornton J.E., LaPierre R.J., Pothoulakis C., Hagan J.P. (2011). Lin28A and Lin28B inhibit let-7 microRNA biogenesis by distinct mechanisms. Cell.

[73] Wu H., Liu B., Chen Z., Li G., Zhang Z. (2020). MSC-induced lncRNA HCP5 drove fatty acid oxidation through miR-3619-5p/AMPK/PGC1α/CEBPB axis to promote stemness and chemo-resistance of gastric cancer. Cell Death Dis..

[74] Tan A., Li Q., Chen L. (2019). CircZFR promotes hepatocellular carcinoma progression through regulating miR-3619–5p/CTNNB1 axis and activating Wnt/β-catenin pathway. Arch. Biochem. Biophys..

[75] Zhang M., Luo H., Hui L. (2019). MiR-3619-5p hampers proliferation and cisplatin resistance in cutaneous squamous-cell carcinoma via KPNA4. Biochemical and biophysical research communications.

[76] Yan G., Su Y., Ma Z., Yu L., Chen N. (2019). Long noncoding RNA LINC00202 promotes tumor progression by sponging miR-3619-5p in retinoblastoma. Cell Struct. Funct..

[77] Ren Z-m, Gu Y.-L., Li X.-M., Zhang Y. (2017). Effect of miR-3619-5p on proliferation and apoptosis of breast cancer cell MCF-7 and T47D and its molecular mechanism. Chin. J. Clin. Exp. Pathol..

[78] Li S., Hu J., Yu X., Xu H., Wang S., Ye Z. (2017). Effect of miR-3619-5p on proliferation of human bladder cancer cell lines EJ and T24. Chinese Journal of Urology.

[79] Sun Z., Li X., Shi Y., Yao Y. (2022).

[80] Bao Y., Cui Y., Luan Y. (2022). Molecular & Cellular Toxicology.

[81] Ma J., Huang L., Gao Y.-B., Li M.-X., Chen L.-L., Yang L. (2022). M2 macrophage facilitated angiogenesis in cutaneous squamous cell carcinoma via circ_TNFRSF21/miR-3619-5p/ROCK axis. Kaohsiung J. Med. Sci..

[82] Liu Y., Li J., Wang S., Song H., Yu T. (2020). STAT4-mediated down-regulation of miR-3619-5p facilitates stomach adenocarcinoma by modulating TBC1D10B. Cancer Biol. Ther..

[83] Li Z., Jiang M., Zhang T., Liu S. (2021). GAS6-AS2 promotes hepatocellular carcinoma via miR-3619-5p/ARL2 axis under insufficient radiofrequency ablation condition. Cancer Biother. Radiopharm..

[84] Liu H., Yin Y., Liu T., Gao Y., Ye Q., Yan J. (2021). Long non-coding RNA PVT1 regulates the migration of hepatocellular carcinoma HepG2 cells via miR-3619-5p/MKL1 axis. Bosn. J. Basic Med. Sci..

[85] Zou Y., Chen B. (2021). Long non-coding RNA HCP5 in cancer. Clinica chimica acta.

[86] Khodayari N., Mohammed K., Goldberg E., Nasreen N. (2011). EphrinA1 inhibits malignant mesothelioma tumor growth via let-7 microRNA-mediated repression of the RAS oncogene. Cancer Gene Ther..

[87] Lin Y., Chen W.M., Wang C., Chen X.Y. (2017). MicroRNA profiling in peripheral T-cell lymphoma, not otherwise specified. Cancer Biomark.

[88] Bai C., Yang W., Ouyang R., Li Z., Zhang L. (2022). Study of hsa_circRNA_000121 and hsa_circRNA_004183 in papillary thyroid microcarcinoma. Open Life Sci..

[89] Wang S., Zhou H., Zhang R., Zhang Y. (2021). Integrated analysis of mutations, miRNA and mRNA expression in glioblastoma. Int. J. Gen. Med..

[90] Shen J.-F., Ge J.-F., Zheng S.-Y., Jiang D. (2021). Integrative analysis of differential circular RNA and long non-coding RNA profiles and associated competing endogenous RNA networks in esophageal squamous cell carcinoma. Funct. Integr. Genom..

[91] Lu P.-W., Li L., Wang F., Gu Y.-T. (2021).

[92] Teeranut A., Saito R., Yasuda J., Tamai K., Miki Y., Abe J. (2022). CANCER SCIENCE.

[93] Wang Y., Zhang B., Zhu Y., Zhang Y., Li L., Shen T. (2022). hsa_circ_0000523/miR-let-7b/METTL3 axis regulates proliferation, apoptosis and metastasis in the HCT116 human colorectal cancer cell line. Oncol. Lett..

[94] Zhang Y., Zhang H., Kang H., Huo W., Zhou Y., Zhang Y. (2019). Knockdown of long non-coding RNA HOST2 inhibits the proliferation of triple negative breast cancer via regulation of the let-7b/CDK6 axis. Int. J. Mol. Med..

[95] Liu Q., Shi H., Yang J., Jiang N. (2019). Long non-coding RNA NEAT1 promoted Hepatocellular Carcinoma cell proliferation and reduced apoptosis through the regulation of Let-7b-IGF-1R Axis. OncoTargets Ther..

[96] Sawan C., Herceg Z. (2010). Histone modifications and cancer. Adv. Genet..

[97] Sadakierska-Chudy A., Filip M. (2015). A comprehensive view of the epigenetic landscape. Part II: histone post-translational modification, nucleosome level, and chromatin regulation by ncRNAs. Neurotox. Res..

[98] Fy Nan, Gu Y., Xu Zj, Sun Gk, Zhou Jd, Zhang Tj (2021). Abnormal expression and methylation of PRR34‐AS1 are associated with adverse outcomes in acute myeloid leukemia. Cancer Med..

[99] Karoopongse E., Yeung C., Byon J., Ramakrishnan A., Holman Z.J., Jiang P.Y.Z. (2014). The KDM2B- let-7b -EZH2 Axis in myelodysplastic syndromes as a target for combined epigenetic therapy. PLoS One.

[100] Zhu Y., Hao F. (2021). Knockdown of long non-coding RNA HCP5 suppresses the malignant behavior of retinoblastoma by sponging miR-3619-5p to target HDAC9. Int. J. Mol. Med..

[101] Zhao Y., Chen Y., Hu X., Zhang N., Wang F. (2020). lncRNA LINC01535 upregulates BMP2 expression levels to promote osteogenic differentiation via sponging miR-3619-5p. Mol. Med. Rep..

[102] Berry N.B., Bapat S.A. (2008). Ovarian cancer plasticity and epigenomics in the acquisition of a stem-like phenotype. J. Ovarian Res..

[103] Brueckner B., Stresemann C., Kuner R., Mund C., Musch T., Meister M. (2007). The human let-7a-3 locus contains an epigenetically regulated microRNA gene with oncogenic function. Cancer Res..

[104] Waly A.A., El-Ekiaby N., Assal R.A., Abdelrahman M.M., Hosny K.A., El Tayebi H.M. (2019). Methylation in MIRLET7A3 gene induces the expression of IGF-II and its mRNA binding proteins IGF2BP-2 and 3 in hepatocellular carcinoma. Front. Physiol..

[105] Heyn H., Vidal E., Ferreira H.J., Vizoso M., Sayols S., Gomez A. (2016). Epigenomic analysis detects aberrant super-enhancer DNA methylation in human cancer. Genome Biol..

[106] Ferreira H.J., Esteller M. (2018). Non-coding RNAs, epigenetics, and cancer: tying it all together. Cancer Metastasis Rev..

[107] Zhang X., Yin Z., Li C., Nie L., Chen K. (2022). KDM2B mediates the Wnt/β-catenin pathway through transcriptional activation of PKMYT1 via microRNA-let-7b-5p/EZH2 to affect the development of non-small cell lung cancer. Exp. Cell Res..

[108] Kuang Y., Xu H., Lu F., Meng J., Yi Y., Yang H. (2021). Inhibition of microRNA let‐7b expression by KDM2B promotes cancer progression by targeting EZH2 in ovarian cancer. Cancer Sci..

[109] Gilles M.-E., Slack F.J. (2018). Let-7 microRNA as a potential therapeutic target with implications for immunotherapy. Expert Opin. Ther. Targets.

[110] Mansoori B., Mohammadi A., Davudian S., Shirjang S., Baradaran B. (2017). The different mechanisms of cancer drug resistance: a brief review. Adv Pharm Bull.

[111] Liu Y., Wang Y., Li X., Jia Y., Wang J., Ao X. (2022). FOXO3a in cancer drug resistance. Cancer Lett..

[112] Qin K, Cheng Y, Zhang J, Yuan X, Wang J, Bai J. Prognostic Risk Model Construction and Prognostic Biomarkers Identification in Esophageal Adenocarcinoma Based on Immune-Related Long Noncoding RNA.

[113] Qian L., Yu B., Chen T., Chen K., Ma Z., Wang Y. (2022). Circ_0022383 alleviates IL-1β-induced apoptosis, inflammation and extracellular matrix degeneration in osteoarthritis cell model by miR-3619-5p/SIRT1 axis. Int. Immunopharm..

[114] Cheng S., Li F., Qin H., Ping Y., Zhao Q., Gao Q. (2022). Long noncoding RNA lncNDEPD1 regulates PD-1 expression via miR-3619-5p in CD8+ T cells. J. Immunol..

[115] Sun B., Han Y., Cai H., Huang H., Xuan Y. (2021). Long non-coding RNA SNHG3, induced by IL-6/STAT3 transactivation, promotes stem cell-like properties of gastric cancer cells by regulating the miR-3619-5p/ARL2 axis. Cell. Oncol..

[116] Wang X., Yu H., Yu Z., Wang D. (2020). Exosomal lncRNA HEIH promotes cisplatin resistance in tongue squamous cell carcinoma via targeting miR-3619-5p/HDGF axis. Acta Histochem..

[117] Wu C., Hu Y., Ning Y., Zhao A., Zhang G., Yan L. (2020). Long noncoding RNA plasmacytoma variant translocation 1 regulates cisplatin resistance via miR-3619-5p/TBL1XR1 axis in gastric cancer. Cancer Biother. Radiopharm..

[118] Ward J.R., Heath P.R., Catto J.W., Whyte M.K., Milo M., Renshaw S.A. (2011). Regulation of neutrophil senescence by microRNAs. PLoS One.

[119] Wang Y., Gu X., Li Z., Xiang J., Jiang J., Chen Z. (2013). microRNA expression profiling in multidrug resistance of the 5-Fu-induced SGC-7901 human gastric cancer cell line. Mol. Med. Rep..

[120] Peng J., Mo R., Ma J., Fan J. (2015). let-7b and let-7c are determinants of intrinsic chemoresistance in renal cell carcinoma. World J. Surg. Oncol..

[121] Han X., Zhang H.-B., Li X.-D., Wang Z.-A. (2020). Long non-coding RNA X-inactive-specific transcript contributes to cisplatin resistance in gastric cancer by sponging miR-let-7b. Anti Cancer Drugs.

[122] Han X., Zhang J.-J., Han Z.-Q., Zhang H.-B., Wang Z.-A. (2018). Let-7b attenuates cisplatin resistance and tumor growth in gastric cancer by targeting AURKB. Cancer Gene Ther..

[123] Ma J., Guo R., Wang T., Pan X., Lei X. (2015). Let-7b binding site polymorphism in the B-cell lymphoma-extra large 3'UTR is associated with fluorouracil resistance of hepatocellular carcinoma. Mol. Med. Rep..

[124] You K., Liu Y., Chen L., Ye H., Lin W. (2022). Radix ranunculus temate saponins sensitizes ovarian cancer to Taxol via upregulation of miR-let-7b. Exp. Ther. Med..

[125] Nishi M., Eguchi-Ishimae M., Wu Z., Gao W., Iwabuki H., Kawakami S. (2013). Suppression of the let-7b microRNA pathway by DNA hypermethylation in infant acute lymphoblastic leukemia with MLL gene rearrangements. Leukemia.

[126] Zhou S., Ma Y., Liu X., Yu P., Huang N., Song L. (2021). Targeted delivery of glypican 3 (GPC3) antibody-modified microRNA (miR let-7b-5p) polymer nanoparticles to sorafenib-resistant hepatsocellular carcinoma cells. J. Biomed. Nanotechnol..

[127] Kumar V., Mondal G., Slavik P., Rachagani S., Batra S.K., Mahato R.I. (2015). Codelivery of small molecule hedgehog inhibitor and miRNA for treating pancreatic cancer. Mol. Pharm..

[128] Lelli D., Pedone C., Sahebkar A. (2017). Curcumin and treatment of melanoma: the potential role of microRNAs. Biomed. Pharmacother..

[129] Klionsky D.J., Petroni G., Amaravadi R.K., Baehrecke E.H., Ballabio A., Boya P. (2021). Autophagy in major human diseases. The EMBO journal.

[130] Liu J., Kuang F., Kroemer G., Klionsky D.J., Kang R., Tang D. (2020). Autophagy-dependent ferroptosis: machinery and regulation. Cell Chem. Biol..

[131] Mathew R., Karantza-Wadsworth V., White E. (2007). Role of autophagy in cancer. Nat. Rev. Cancer.

[132] Lee J.W., Jeong E.G., Lee S.H., Yoo N.J., Lee S.H. (2007). Somatic mutations of BECN1, an autophagy‐related gene, in human cancers. Apmis.

[133] Cao Z., Guan L., Yu R., Chen J. (2022). Identifying autophagy-related lncRNAs and potential ceRNA networks in NAFLD. Front. Genet..

[134] Pawar K., Sharbati J., Einspanier R., Sharbati S. (2016). Mycobacterium bovis BCG interferes with miR-3619-5p control of cathepsin S in the process of autophagy. Front. Cell. Infect. Microbiol..

[135] Liao C.-C., Ho M.-Y., Liang S.-M., Liang C.-M. (2018). Autophagic degradation of SQSTM1 inhibits ovarian cancer motility by decreasing DICER1 and AGO2 to induce MIRLET7A-3P. Autophagy.

[136] Liao C.-C., Ho M.-Y., Liang S.-M., Liang C.-M. (2013). Recombinant protein rVP1 upregulates BECN1-independent autophagy, MAPK1/3 phosphorylation and MMP9 activity via WIPI1/WIPI2 to promote macrophage migration. Autophagy.

[137] Ham O., Lee S.-Y., Lee C.Y., Park J.-H., Lee J., Seo H.-H. (2015). let-7b suppresses apoptosis and autophagy of human mesenchymal stem cells transplanted into ischemia/reperfusion injured heart 7by targeting caspase-3. Stem Cell Res. Ther..

[138] Khurana E., Fu Y., Chakravarty D., Demichelis F., Rubin M.A., Gerstein M. (2016). Role of non-coding sequence variants in cancer. Nat. Rev. Genet..

[139] Sun Q., Gu H., Zeng Y., Xia Y., Wang Y., Jing Y. (2010). Hsa‐mir‐27a genetic variant contributes to gastric cancer susceptibility through affecting miR‐27a and target gene expression. Cancer Sci..

[140] Akella M., Amajala K.C., Malla R.R. (2019). Bioinformatics analysis of regulatory elements of the CD151 gene and insilico docking of CD151 with diallyl sulfide. Gene Reports.

[141] Wang D.J., Legesse-Miller A., Johnson E.L., Coller H.A. (2012). Regulation of the let-7a-3 promoter by NF-κB. PLoS One.

[142] Balzeau J., Menezes M.R., Cao S., Hagan J.P. (2017). The LIN28/let-7 pathway in cancer. Front. Genet..

[143] Liu C-y, Stücker I., Chen C., Goodman G., McHugh M.K., D'Amelio A.M. (2015). Genome-wide gene–asbestos exposure interaction association study identifies a common susceptibility variant on 22q13. 31 associated with lung cancer RiskGenome-wide gene–asbestos interaction and lung cancer risk. Cancer Epidemiol. Biomarkers Prev..

[144] Abdi E., Latifi-Navid S., Kholghi-Oskooei V., Pourfarzi F., Yazdanbod A. (2021). Interaction between lncRNAs HOTAIR and MALAT1 tagSNPs in gastric cancer. Br. J. Biomed. Sci..

[145] Patrão A.S., Dias F., Teixeira A.L., Maurício J., Medeiros R. (2018). XPO5 genetic polymorphisms in cancer risk and prognosis. Pharmacogenomics.

[146] Malek E., Jagannathan S., Driscoll J.J. (2014). Correlation of long non-coding RNA expression with metastasis, drug resistance and clinical outcome in cancer. Oncotarget.

[147] Yamamoto Y., La Salvia S., Susmita S., Tahara H. (2021). Potential Biomarkers for Therapeutic Monitoring and Clinical Outcome in Breast Cancer, Breast Cancer-Evolving Challenges and Next Frontiers.

[148] Wang X., Ye L., Li B. (2022). Development of a genomic instability-derived lncRNAs-based risk signature as a predictor of prognosis for endometrial cancer. J. Cancer.

[149] Zi H., Tuo Z., He Q., Meng J., Hu Y., Li Y. (2022). Comprehensive bioinformatics analysis of gasdermin family of glioma. Comput. Intell. Neurosci..

[150] Yu S., Cao S., Hong S., Lin X., Guan H., Chen S. (2019). miR-3619-3p promotes papillary thyroid carcinoma progression via Wnt/β-catenin pathway. Ann. Transl. Med..

[151] Si L., Chen J., Yang S., Liu Z., Chen Y., Peng M. (2020). lncRNA HEIH accelerates cell proliferation and inhibits cell senescence by targeting miR-3619-5p/CTTNBP2 axis in ovarian cancer. Menopause.

[152] Sun Y., Liu Y., Cai Y., Han P., Wang R., Cao L. (2020). Downregulation of LINC00958 inhibits proliferation, invasion and migration, and promotes apoptosis of colorectal cancer cells by targeting miR-3619-5p. Oncol. Rep..

[153] Li S., Wang C., Yu X., Wu H., Hu J., Wang S. (2017). miR-3619-5p inhibits prostate cancer cell growth by activating CDKN1A expression. Oncol. Rep..

[154] Hu K., Li N.F., Li J.R., Chen Z.G., Wang J.H., Sheng L.Q. (2021). Exosome circCMTM3 promotes angiogenesis and tumorigenesis of hepatocellular carcinoma through miR‐3619‐5p/SOX9. Hepatol. Res..

[155] Chen X., Zhao S., Li Q., Xu C., Yu Y., Ge H. (2020). LncRNA NEAT1 knockdown inhibits retinoblastoma progression by miR-3619-5p/LASP1 axis. Front. Genet..

[156] Song B., Li H., Guo S., Yang T., Li L., Cao L. (2022). LINC00882 plays a tumor-promoter role in colorectal cancer by targeting miR-3619-5p to up-regulate CTNNB1. Arch. Med. Res..

[157] Zhang D., Gu G., Chen X., Zha G., Yuan Z., Wu Y. (2020). LINC00665 facilitates the progression of osteosarcoma via sponging miR-3619-5p. Eur. Rev. Med. Pharmacol. Sci..

[158] Lv M., Mao Q., Li J., Qiao J., Chen X., Luo S. (2020). Knockdown of LINC00665 inhibits proliferation and invasion of breast cancer via competitive binding of miR-3619-5p and inhibition of catenin beta 1. Cellular & molecular biology letters.

[159] Xia H., Niu Q., Ding Y., Zhang Z., Yuan J., Jin W. (2021). Long noncoding HOXA11-AS knockdown suppresses the progression of non-small cell lung cancer by regulating miR-3619-5p/SALL4 axis. J. Mol. Histol..

[160] Zhang Q., Miao S., Han X., Li C., Zhang M., Cui K. (2018). MicroRNA-3619-5p suppresses bladder carcinoma progression by directly targeting β-catenin and CDK2 and activating p21. Cell Death Dis..

[161] Wu Sr B., Pan Sr Y., Jin Y., Zhao M., Zhou F. (2018). Analysis of differential circrnas expression profile identifies novel biomarkers for acute monocytic leukemia. Blood.

[162] Tanaka S., Hosokawa M., Matsumura J., Matsubara E., Kobori A., Ueda K. (2017). Effects of zebularine on invasion activity and intracellular expression level of let-7b in colorectal cancer cells. Biol. Pharm. Bull..

[163] Saffari M., Ghaderian S.M.H., Omrani M.D., Afsharpad M., Shankaie K., Samadaian N. (2019). The association of miR-let 7b and miR-548 with PTEN in prostate cancer. Urol. J..

[164] Gu S.-Y., Zhang G.P., Si Q., Dai J., Song Z., Wang Y. (2021). Web tools to perform long non-coding RNAs analysis in oncology research. Database: The Journal of Biological Databases and Curation.

[165] Kim T., Croce C.M. (2023). MicroRNA: trends in clinical trials of cancer diagnosis and therapy strategies. Experimental & molecular medicine.

[166] Solomon J., Kern F., Fehlmann T., Meese E., Keller A. (2020). HumiR: web services, tools and databases for exploring human microRNA data. Biomolecules.

[167] Liu C.J., Fu X., Xia M., Zhang Q., Gu Z., Guo A.Y. (2021). miRNASNP-v3: a comprehensive database for SNPs and disease-related variations in miRNAs and miRNA targets. Nucleic Acids Res..

